# Structure and Spectroscopy of Triruthenium Dodecacarbonyl, Ru_3_(CO)_12_

**DOI:** 10.1021/acs.inorgchem.5c05397

**Published:** 2026-01-05

**Authors:** Stewart F. Parker, A. Dominic Fortes

**Affiliations:** ISIS Neutron and Muon Facility, 97008 STFC Rutherford Appleton Laboratory, Harwell Science and Innovation Campus, Chilton, Oxfordshire OX11 0QX, U.K.

## Abstract

We have reinvestigated the structure and vibrational spectroscopy of triruthenium dodecacarbonyl, Ru_3_(CO)_12_, in the solid state by neutron powder diffraction, inelastic neutron scattering (INS), infrared and Raman spectroscopies. We find that the known room temperature *P*2_1_/*n* structure is maintained down to 10 K. The unit-cell parameters follow a Debye relationship, with an overall 5.9% volume contraction from 300 to 10 K. The high resolution data at 10 K has enabled the most precise determination of the structure to date. INS spectroscopy, which has no selection rules, has allowed the observation of all the fundamentals below 700 cm^–1^ for the first time. In combination with new low temperature infrared and Raman spectra, this has resulted in the reassignment of several of the modes. Our previous work has shown that density functional theory calculations of the vibrational spectra of metal carbonyls produces mixed results. For M­(CO)_6_, M = Cr, Mo, W the calculated spectra are in good agreement with the experimental spectra, whereas for Fe­(CO)_5_, and Fe_2_(CO)_9_ the agreement was poor. We find that for Ru_3_(CO)_12_ the agreement is also poor.

## Graphical abstract

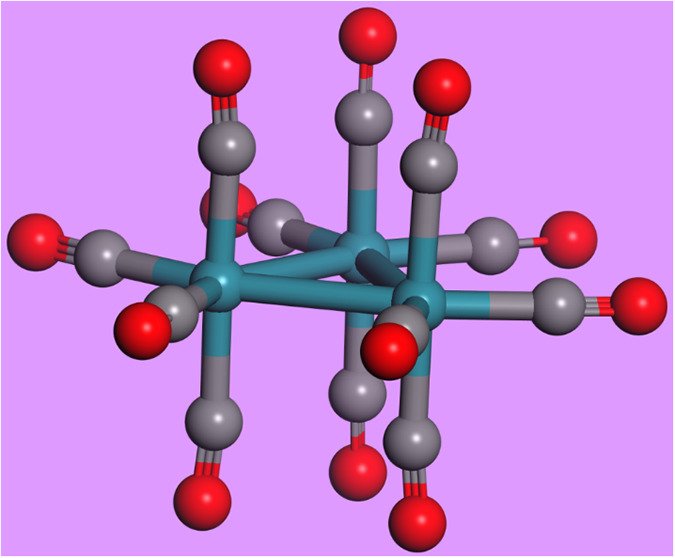

## Introduction

In 1910 Mond, Hirtz, and Cowap^1^ reported that the reaction of ruthenium black with carbon monoxide at 350–450 bar and 300 °C resulted in the formation of a small amount of a yellow-orange solid that was believed to be a ruthenium carbonyl. Subsequent work by Manchot and Manchot^2^ using even more forcing conditions (700 bar and 400 °C) produced a yellow liquid that decomposed to an orange solid. The yellow liquid was identified as Ru­(CO)_5_ and the orange solid as ruthenium enneacarbonyl, Ru_2_(CO)_9_. It was only in 1961 that Corey and Dahl^3^ correctly identified the orange compound as triruthenium dodecacarbonyl, Ru_3_(CO)_12_, by its isomorphism to Os_3_(CO)_12_, whose structure had been determined by single crystal X-ray diffraction. Subsequent more comprehensive studies^4–7^ confirmed the result.

The structure of Ru_3_(CO)_12_ is shown in Figure 1, in solution the symmetry is *D*_3h_, in the solid state it is formerly *C*_1_, but is actually very close to *D*_3h_. The development of methods that allowed large quantities of Ru_3_(CO)_12_ to be prepared^8^ enabled the vibrational spectroscopy to be investigated^7,9–16^ and its chemistry to be explored. Ru_3_(CO)_12_ has an extensive photochemistry,^17–19^ it finds use as a homogeneous catalyst for a variety of reactions^20–24^ and also as a precursor for supported Ru catalysts.^25–27^

**Figure 1:**
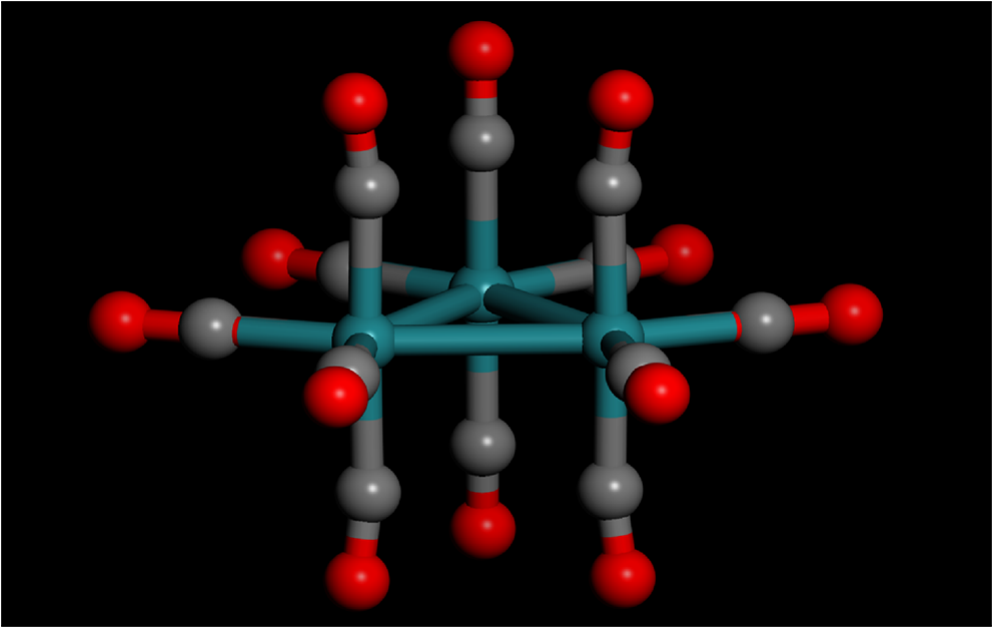
Idealised *D*_3h_ structure of Ru_3_(CO)_12_. Key: green = Ru, gray = C, red = O.

Despite the extensive work on both the structure and the spectroscopy, there are still some ambiguities. The room temperature structure is maintained down to 100 K but whether there are any phase transitions below this is unknown. While the C≡O stretch region has been comprehensively assigned, the assignment of the 0–800 cm^–1^ region, where the Ru–C stretch, Ru–C≡O deformation, C–Ru–C bend and Ru–Ru stretch modes occur is much less certain because several of the modes exhibit negligible intensity.

Neutron scattering potentially provides answers to both of these uncertainties. Neutron diffraction^28^ data is readily obtained down to limiting low temperatures. In addition to detecting phase transitions, it may also provide very accurate and precise bond distances and angles. Neutron vibrational spectroscopy^29^ (inelastic neutron scattering, INS) has no selection rules and all the modes are, in principle, observable. The resolution in the C≡O stretch region is insufficient to resolve the modes but in the region below 800 cm^–1^, the resolution is comparable to routine infrared and Raman measurements. In previous work we have used the combination of neutron powder diffraction (NPD) and INS to observe a new low-temperature phase of Fe­(CO)_5_ ^30^ and to provide the first complete assignment of the low energy modes. INS has also been used to assign the low energy modes of the metal hexacarbonyls, M­(CO)_6_, (M = Cr, Mo, W)^31^ and of Fe_2_(CO)_9_.^32^

Vibrational spectroscopy assignments are usually supported by density functional theory (DFT) calculations.^29,33,34^ For the hexacarbonyls, periodic-DFT calculations of the solid state spectra were in good agreement with the experimental data. For Fe­(CO)_5_ ^30^ and Fe_2_(CO)_9_ ^32^ this was not the case and both isolated molecule and fully periodic calculations gave poor agreement. It is of interest to see how well DFT performs for Ru_3_(CO)_12_.

In this work, we have determined the solid-state structure by neutron powder diffraction between 10 and 300 K. We have measured the INS spectrum at ∼10 K. To update the conventional infrared and Raman spectra, we have recorded Raman spectra in the range 7–300 K and infrared spectra between 173 and 300 K. We have also investigated the performance of DFT calculations for this system.

## Results and Discussion

### Neutron Powder Diffraction

The room temperature structure of Ru_3_(CO)_12_ is monoclinic, space group *P*2_1_/*n* (no. 14)^3–6^ and it is known that this is maintained down to at least 100 K.^6^ In the present work we have measured the lattice parameters from 300 to 10 K. The temperature dependence of the unit-cell parameters and the unit-cell volume of Ru_3_(CO)_12_ are shown in Figure 2 and the values reported in Table S1. The values obtained at 300, 150, and 100 K, are within errors, the same as measured by Churchill et al.^5^ and Braga et al.^6^ It is apparent that the *P*2_1_/*n* structure persists throughout the 10–300 K interval. Note that the unique monoclinic angle, β, varies by less than 0.2° on cooling close to absolute zero.

**Figure 2:**
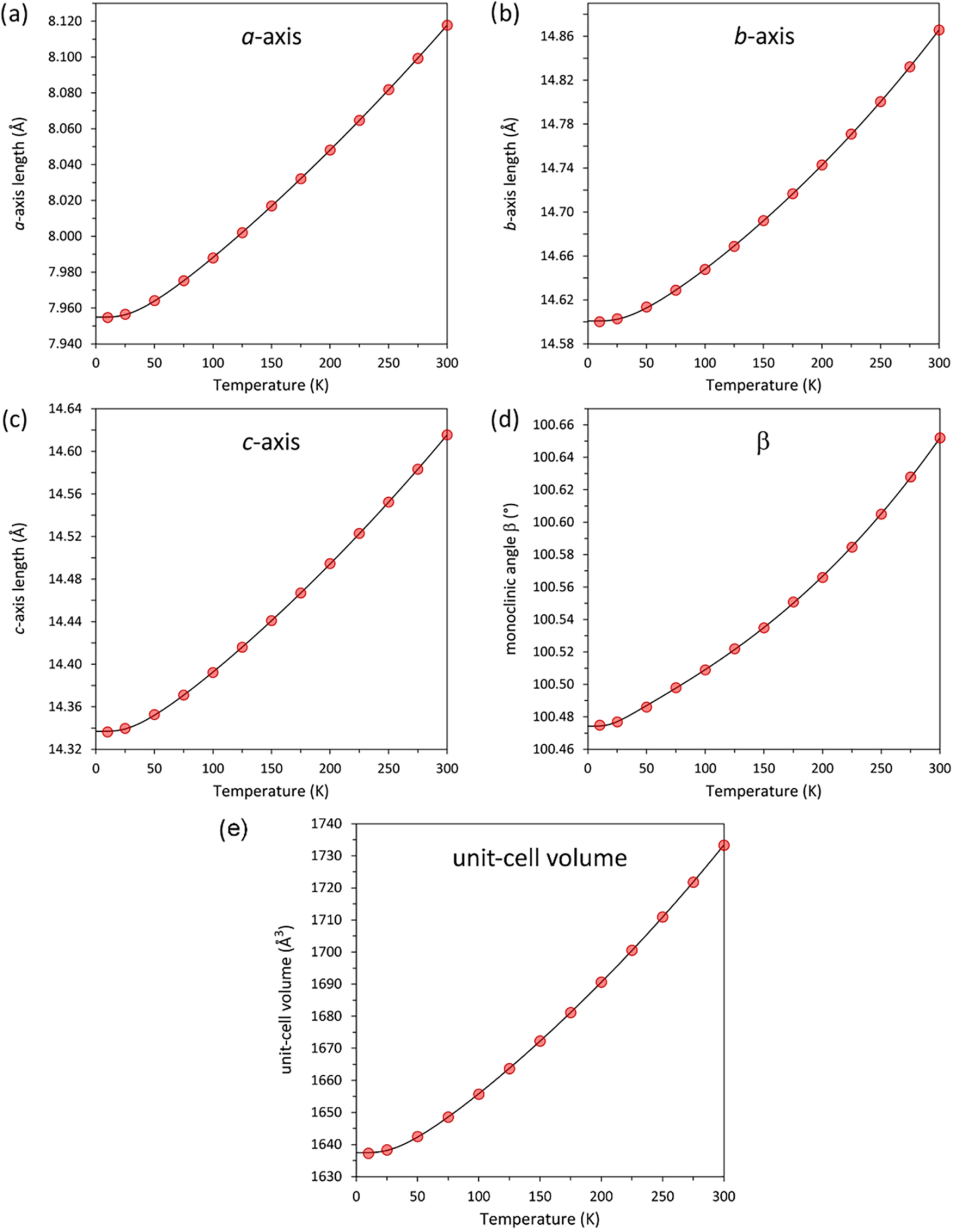
Temperature dependence of the unit-cell parameters of Ru_3_(CO)_12_ from neutron powder diffraction data collected on warming from 10 to 300 K. It is apparent that there are no phase transitions. (a) *a*-axis, (b) *b*-axis, (c) *c*-axis, (d) β angle and (e) unit-cell volume.

The unit-cell volume decreases on cooling from 300 to 10 K by 5.5%. A high pressure study^7^ of Ru_3_(CO)_12_ found that it was highly compressible, decreasing in molar volume by 25% between atmospheric pressure and 8.14 GPa. The drop in volume was the result of a reduction in the intermolecular space, rather than structural changes. Comparison of the room temperature and 10 K structural parameters, Table S1, shows that this is also the case on cooling. The unit-cell volume of Ru_3_(CO)_12_ has been fitted with a second-order Grüneisen approximation to the zero-pressure equation of state.^35^ In this approximation, the thermal expansion is considered equivalent to elastic strain such that

1 $$V\left(T\right)=V_{0}\left[1+\frac{E\left(T\right)}{Q-\mathrm{bE}\left(T\right)}\right]$$

where *V*_0_ is the unit cell volume at zero pressure, *b* = 1/2­(*K*′_0_ – 1) and *Q* = (*V*_0_ *K*_0_/γ); *K*_0_ is the zero-pressure isothermal bulk modulus, *K*′_0_ is its first derivative with respect to pressure, and γ is the thermal Grüneisen parameter. The internal energy due to lattice vibrations, *E*(*T*), is then determined via a simple Debye model approximation of the phonon density of states

2 $$E\left(T\right)=\frac{9nk_{B}T}{{\left(\theta_{D}/T\right)}^{3}}\int_{0}^{\theta_{D}/T}\frac{x^{3}}{e^{x}-1}dx$$

where θ_D_ is the Debye temperature, *n* is the number of atoms per formula unit, and *k*_B_ is the Boltzmann constant; the integral term is evaluated numerically.

A similar method was adopted to fit the unit-cell parameters. However, in order to be formally dimensionally correct, the parameters have each been cubed and the temperature dependence of the monoclinic angle has been derived from the fitting of both *a* ^3^ and (*a·*sin β)^3^.

Table 1 reports the parameters obtained from fitting eq 1 to the cubed unit-cell parameters and the unit-cell volume of Ru_3_(CO)_12_. The resulting fits to the Debye-type model are shown as the solid lines in Figure 2.

**Table 1:** Parameters Obtained by the Fitting of a Debye-Type Model to the Unit-Cell Volume of Ru_3_(CO)_12_ as a Function of Temperature (Equations 1 and 2)

	(*a*-axis)^3^	(*b*-axis)^3^	(*c*-axis)^3^	(*a·*sin β)^3^	volume
θ_D_ (K)	135.7(4)	146.2(7)	132.9(6)	138.1(5)	137.8(5)
*V*_0_ (cm^3^ mol^–1^)	75.7875(6)	468.621(5)	443.667(4)	72.0617(6)	246.524(2)
*Q* (J cm^–3^)	3.223(4) × 10^6^	4.085(9) × 10^6^	3.537(6) × 10^6^	3.271(5) × 10^6^	3.617(6) × 10^6^
*b*	3.02(2)	6.35(3)	3.92(3)	2.88(2)	4.29(2)
Debye cutoff (cm^–1^)	94.3(3)	101.6(5)	92.4(4)	96.0(3)	95.8(3)
*V*_0_ (Å^3^)	503.392(9)	3112.65(6)	2946.90(6)	478.645(9)	1637.45(3)
*K*_0_/γ (GPa)	42.53(6)	8.72(2)	7.97(1)	45.39(7)	14.67(2)
*K*′_0_	7.05(4)	13.71(7)	8.85(5)	6.76(4)	9.57(5)

In Table 1, lines 1 to 4 report parameters obtained by the fitting of a Debye-type model to the unit-cell volume of Ru_3_(CO)_12_ as a function of temperature (eqs 1 and 2). The last four rows of the table report values derived from the parameters in the preceding rows. Note that the Debye temperatures obtained from fitting each direction in the crystal are very similar; however, the elastic stiffness of the *a*-axis is substantially higher than that of either the *b*- or *c*-axis. This likely reflects the packing of the trimers into layers, the direction perpendicular to the layers being less compressible.

Eulerian infinitesimal strain tensors were calculated from pairs of cell parameters determined at adjacent temperatures and then normalized by the temperature increment between them in order to obtain thermal expansion tensors, *i.e*., unit-strain tensors.^36^ Standard matrix decomposition methods^37^ were used to derive the eigenvalues and eigenvectors of the thermal expansion tensor, these being the magnitudes and orientations of the principal expansivities.

The temperature dependences of the three principal linear expansivities are shown in Figure 2, and the sum of these three components–the volume thermal expansion–is plotted in Figure 2. The same analysis was carried out on values derived from the Debye model fits, as represented by the solid lines on Figures 3 and 4.

**Figure 3:**
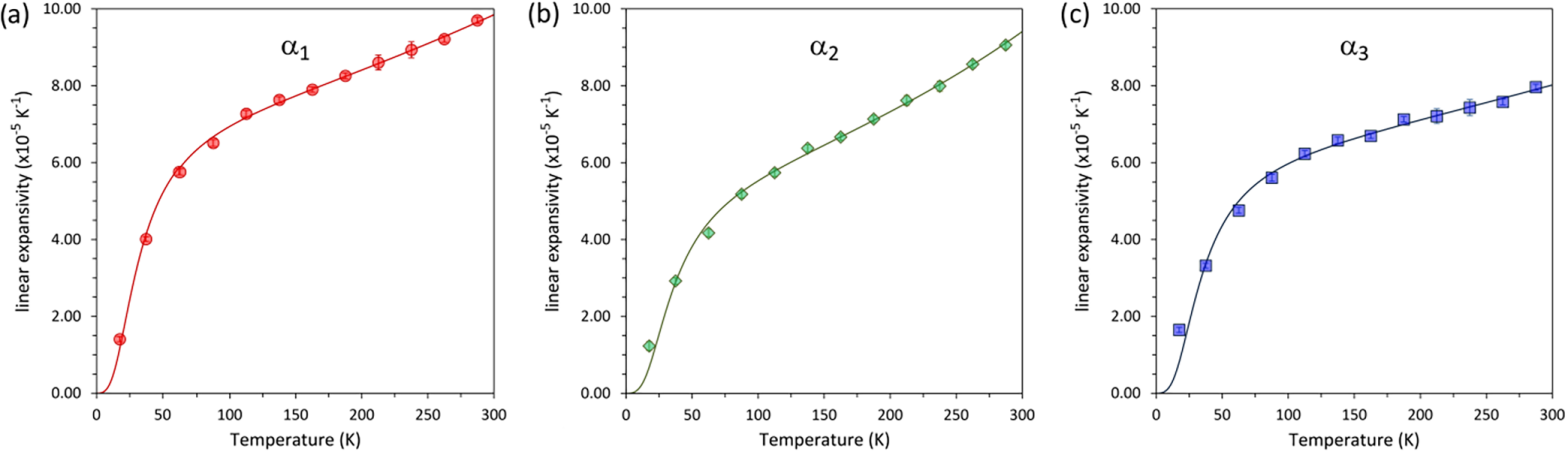
Principal thermal expansivities as a function of temperature: (a) α_1_; (b) α_2_; (c) α_3_.

**Figure 4:**
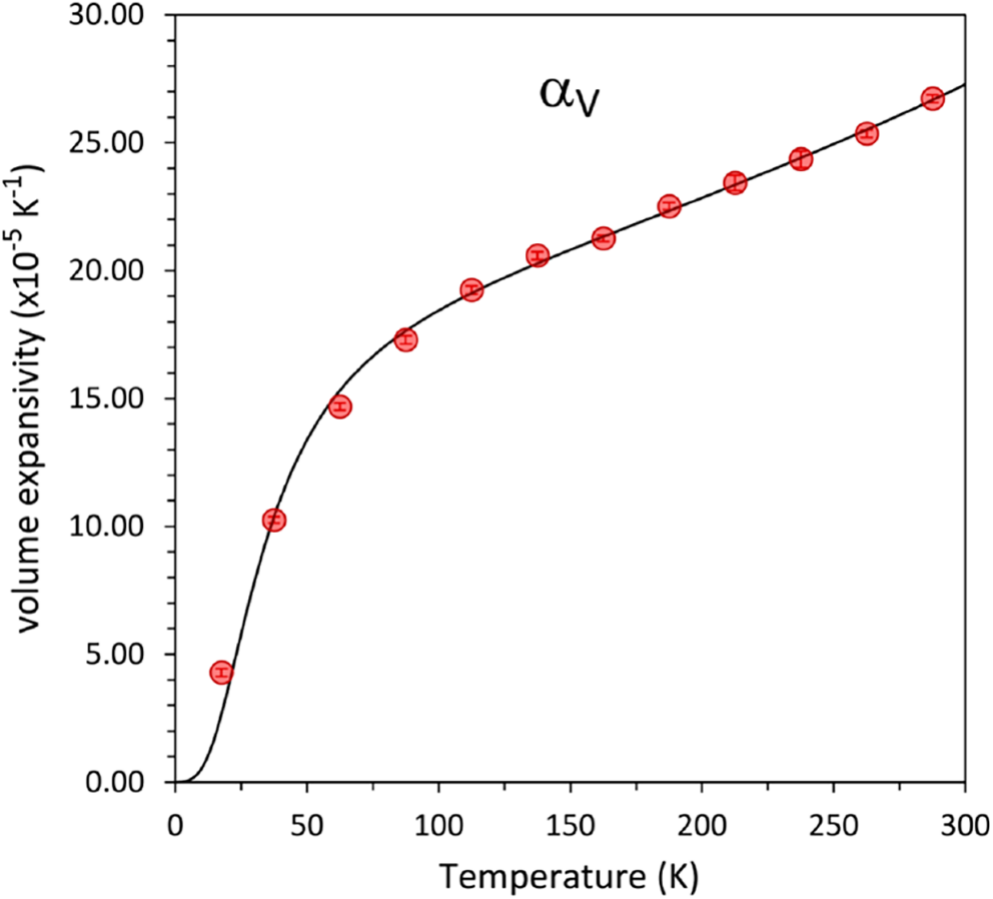
Volume thermal expansion of Ru_3_(CO)_12_.

The thermal expansion tensor shown in Figure 5 is depicted by a representation surface,^38^ oriented in relation to the orthogonal basis (*x*,*y*,*z*) used in its derivation. These directions correspond with the crystallographic basis as, x||*a·*sin β, *y*||*b*, and *z*||*c*. Note that the thermal expansion shown (at 300 K) is comparatively isotropic, which remains true upon cooling to 10 K, there being only slight variations in the orientation of the principal axes.

**Figure 5:**
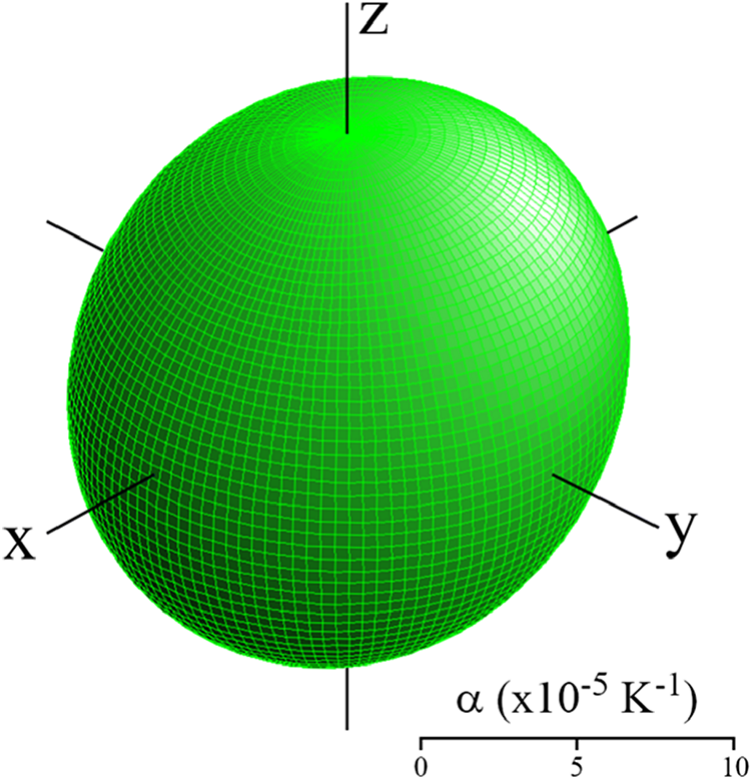
Thermal expansion tensor of Ru_3_(CO)_12_ at 300 K. Drawn using WinTensor.^39^

Being a monoclinic crystal, the principal direction α_2_ is constrained by symmetry to coincide with the 2-fold axis, while α_1_ and α_3_ need not be aligned with any particular crystallographic direction. The orientation of these vectors with respect to the crystallographic basis may be represented by one angle, for example the angle between α_1_ and *x* (or *a·*sin β). The temperature dependence of this quantity is shown in Figure 6.

**Figure 6:**
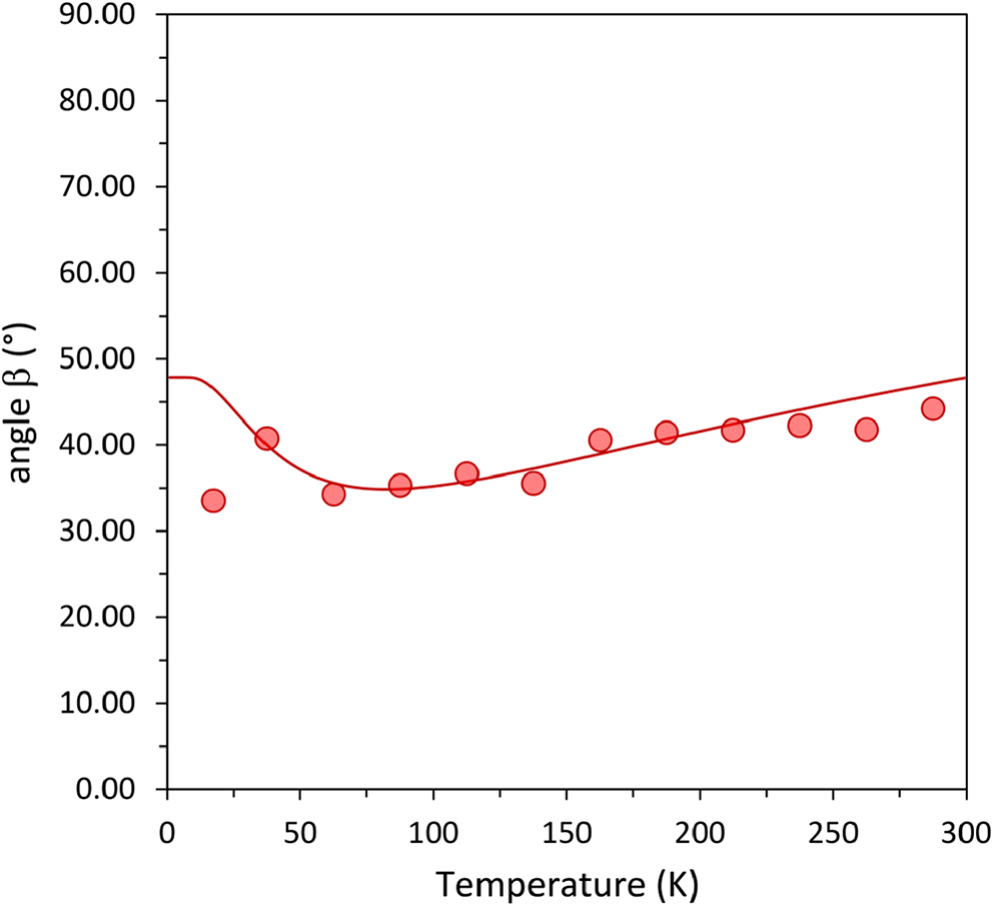
Angle between the principal expansion direction α_1_ and the orthogonal basis vector *x* as a function of temperature. The symbols are derived from the data in Table 1 and the solid line from the fitting of a Debye-type model.

The structure of Ru_3_(CO)_12_ at 10 K is shown in Figure S1. The site symmetry is *C*_1_, so there are no requirements for equivalent bond distances and angles to be the same. Nonetheless, as can be seen from Figure S1, the structure is very close to *D*_3h_. Table 2 lists averaged bond lengths and angles from previous work and our results. These confirm the conclusions of Churchill et al.^5^ namely that all of the C≡O bond distances are the same, that the axial Ru–C bonds are longer than the equatorial ones, axial Ru–C≡O bonds are bent by ∼7°, that the equatorial ones are essentially linear and that the Ru_3_ triangle is very close to being an equilateral triangle.

**Table 2:** Comparison of Intramolecular Bond Lengths in Ru_3_(CO)_12_ between Our Work and Values Reported in the Literature

	RT^5^	100 K^6^	10 K^a^	CASTEP^a^,^b^	DMol^3^ ^a^,^b^	Gaussian^a^,^c^
distance (Å)						
Ru–Ru	2.851, 2.852, 2.860	2.844, 2.846, 2.859	2.842, 2.849, 2.855	2.929, 2.929, 2.927	2.882, 2.887, 2.897	2.921, 2.921, 2.921
av. C≡O_ax_	1.133	1.137	1.138	1.143	1.156	1.174
av. C≡O_eq_	1.127	1.136	1.138	1.142	1.156	1.175
av. Ru–C_ax_	1.942	1.951	1.950	1.980	1.950	1.959
av. Ru–C_eq_	1.921	1.925	1.930	1.951	1.917	1.933
bond angle (deg)						
Ru–Ru–Ru			60.23, 60.03, 59.74	60.18, 59.92, 59.90	60.174, 59.998, 59.828	60.00, 60.00, 60.00
av. Ru–C≡O_ax_		173.3	173.45	171.28	172.81	171.64
av. Ru–C≡O_eq_		178.6	178.60	178.68	178.87	178.49

a This work.

b Periodic calculation of the *P*2_1_/*c* primitive cell.

c Isolated molecule calculation with *D*_3h_ symmetry imposed.

### Vibrational Spectroscopy

Idealised Ru_3_(CO)_12_ has *D*_3h_ symmetry and the distribution of the modes and their activity has been given by Adams and Taylor,^13^ their analysis is reproduced in Table 3. There are no symmetry operations that interchange the axial and equatorial carbonyls, so they are treated as distinct entities. Note that under *D*_3h_ symmetry, 10 of the 50 modes are forbidden in both the infrared and Raman spectra. In the solid state, the low site symmetry of *C*_1_ means that all degeneracies are lifted and all modes are allowed. The presence of four molecules in the primitive cell results in every mode having two infrared allowed and two Raman allowed factor group components.^40^ However, while the *D*_3h_ forbidden modes are now formally allowed, the selection rules do not give any indication of intensity (a mode can be allowed but have essentially zero intensity), they are likely to have very low infrared and Raman intensities. Note that *all* modes are allowed in the INS.

**Table 3:** Ru_3_(CO)_12_ in *D*_3h_ Symmetry: Distribution of the Modes, Their Activity, Transition Energies (cm^–1^) and Assignments^a^

sym	mode no.	description	activity	Quicksall^9^	Kettle^11^	Adams^13^	this work
*A*′_1_	ν_1_	ν(C≡O)_ax_	R		2120	2120	2120
*A*′_1_	ν_2_	ν(C≡O)_eq_	R		2036	2028	2028
*A*′_1_	ν_3_	ν(Ru–CO)_ax_	R			459	457
*A*′_1_	ν_4_	ν(Ru–CO)_eq_	R			383	432
*A*′_1_	ν_5_	δ(Ru–C≡O)	R			449	588
*A*′_1_	ν_6_	δ(Ru–C≡O)	R			489	490
*A*′_1_	ν_7_	ν(Ru–Ru)	R	189	187	185	185
*A*′_1_	ν_8_	δ(OC–Ru–CO)	R	85		124	142
*A*′_1_	ν_9_	δ(OC–Ru–CO)	R	47		86	82
*A*′_2_	ν_10_	ν(C≡O)_eq_	inactive		2000	1990	
*A*′_2_	ν_11_	ν(Ru–CO)_eq_	inactive				566
*A*′_2_	ν_12_	δ(Ru–C≡O)	inactive				430
*A*′_2_	ν_13_	δ(Ru–C≡O)	inactive				390
*A*′_2_	ν_14_	δ(OC–Ru–CO)	inactive				91
*A*′_2_	ν_15_	δ(OC–Ru–CO)	inactive				48
*E*′	ν_16_	ν(C≡O)_ax_	IR and R		2061	2060	2064
*E*′	ν_17_	ν(C≡O)_eq_	IR and R		2019	2019	2021
*E*′	ν_18_	ν(C≡O)_eq_	IR and R		2012	1996	2006
*E*′	ν_19_	ν(Ru–CO)_ax_	IR and R			449	442
*E*′	ν_20_	ν(Ru–CO)_eq_	IR and R			403	420
*E*′	ν_21_	ν(Ru–CO)_eq_	IR and R			391	382
*E*′	ν_22_	δ(Ru–C≡O)	IR and R			604	603/610
*E*′	ν_23_	δ(Ru–C≡O)	IR and R			580	578/580
*E*′	ν_24_	δ(Ru–C≡O)	IR and R			548	471
*E*′	ν_25_	δ(Ru–C≡O)	IR and R			515	442
*E*′	ν_26_	ν(Ru–Ru)	IR and R	149	151	151	151
*E*′	ν_27_	δ(OC–Ru–CO)	IR and R			131	106
*E*′	ν_28_	δ(OC–Ru–CO)	IR and R	100		116	95
*E*′	ν_29_	δ(OC–Ru–CO)	IR and R			106/101	75
*E*′	ν_30_	δ(OC–Ru–CO)	IR and R			90	57
*A*″_1_	ν_31_	δ(Ru–C≡O)	inactive				513
*A*″_1_	ν_32_	δ(Ru–C≡O)	inactive				390
*A*″_1_	ν_33_	δ(OC–Ru–CO)	inactive				89
*A*″_1_	ν_34_	δ(OC–Ru–CO)	inactive				20
*A*″_2_	ν_35_	ν(C≡O)_ax_	IR		2031	2041	2044
*A*″_2_	ν_36_	ν(Ru–CO)_ax_	IR			435	543
*A*″_2_	ν_37_	δ(Ru–C≡O)	IR			468	442
*A*″_2_	ν_38_	δ(Ru–C≡O)	IR			449	410
*A*″_2_	ν_39_	δ(OC–Ru–CO)	IR			140	141
*A*″_2_	ν_40_	δ(OC–Ru–CO)	IR			68	88
*E*″	ν_41_	ν(C≡O)_ax_	R		1994	1986	1987
*E*″	ν_42_	ν(Ru–CO)_ax_	R			420	544
*E*″	ν_43_	δ(Ru–C≡O)	R				512
*E*″	ν_44_	δ(Ru–C≡O)	R				431
*E*″	ν_45_	δ(Ru–C≡O)	R				399
*E*″	ν_46_	δ(Ru–C≡O)	R				390
*E*″	ν_47_	δ(OC–Ru–CO)	R	124		79	111
*E*″	ν_48_	δ(OC–Ru–CO)	R				93
*E*″	ν_49_	δ(OC–Ru–CO)	R				76
*E*″	ν_50_	δ(OC–Ru–CO)	R				44

a ax = axial, eq = equatorial, ν = stretch, δ = bend, R= Raman allowed, IR = infrared allowed.

Figure 7 shows the vibrational spectra of Ru_3_(CO)_12_ in the solid state in the low energy region, which is the focus of the present study, the observed bands are given in Table 3. (The complete spectra 0–2200 cm^–1^ are shown in Figure S2). We defer a detailed assignment of the spectra to the next section, where we support these with DFT calculations. However, several points are worth noting.

**Figure 7:**
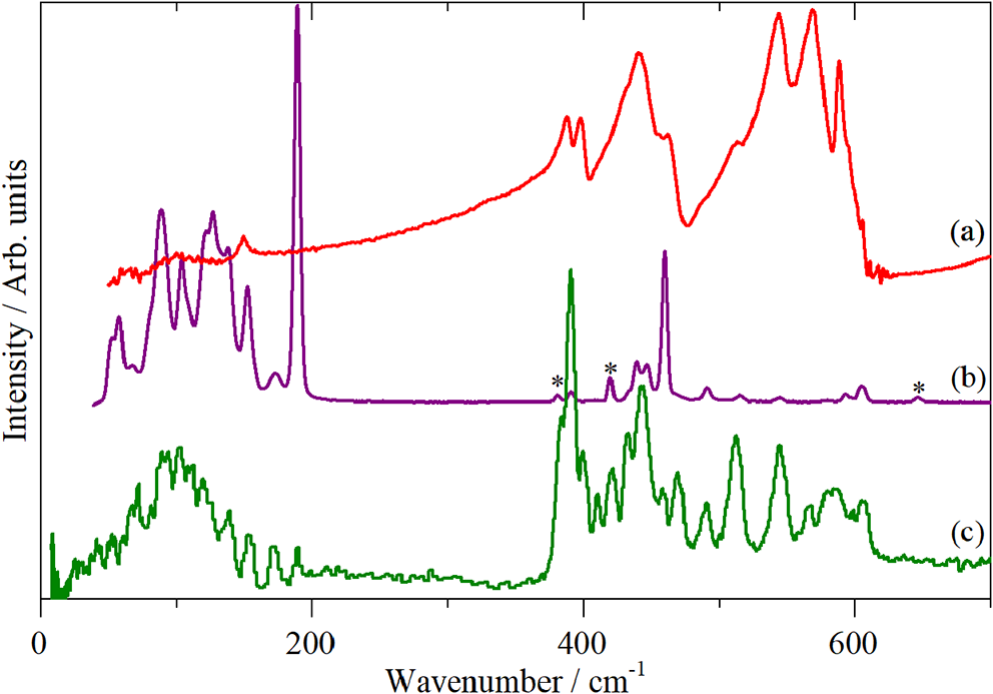
Vibrational spectra of Ru_3_(CO)_12_. (a) Infrared at 296 K, (b) Raman (785 nm) at 7 K and (c) INS at 10 K. In (b) the asterisks denote bands due to the sapphire window of the sample holder.

In the 300–700 cm^–1^ region 24 modes are expected. In the INS spectrum, there are, at most, only 17 modes apparent. As all modes are allowed and must be present, it immediately follows that there must be at least seven accidental degeneracies. We note that while overtones and combinations are allowed transitions in INS spectroscopy and for hydrogenous systems can be almost as intense as some fundamentals. However, their intensity depends on the mass of the atom in the mode and for non-hydrogenous systems the intensity is negligible. This is advantageous as it means that any modes of reasonable intensity in the INS spectrum will be fundamentals.

As expected, there are no bands in the 200–360 cm^–1^ interval because there are no bridging carbonyls present.

In the region below 200 cm^–1^, in addition to the OC–Ru–CO bending modes and the Ru–Ru stretch modes, there are three acoustic translational modes, nine optic translational modes and 12 librational modes. This results in the very complex feature seen in the INS spectrum. As both the translations and the librations require the entire molecule to move, these are likely to form the low energy side of the feature below 100 cm^–1^ and the internal modes forming the 100–200 cm^–1^ bands. All of the INS features across the 100–800 cm^–1^ interval are surprisingly sharp which would suggest that there is no significant vibrational dispersion present (variation of transition energy with wavevector). INS is sensitive to all wavevectors, in contrast to infrared and Raman spectroscopy that are seen at zero wavevector, As the INS spectrum can be considered to be proportional to the projection of the vibrational dispersion curves onto the energy axis, any significant dispersion would results in broadened bands, perhaps with unusual lineshapes. It follows that as the bandwidths are not much larger than the instrument resolution, the dispersion must be small, 10 cm^–1^ or so.

### Computational Studies and Assignment of the Spectra

The C≡O stretch region of Ru_3_(CO)_12_ has been investigated by several groups^11,13–15^ and there is general agreement on the assignments. Our infrared and Raman spectra are in good agreement with those in the literature and the INS does not provide any useful information in this region, so we have adopted the literature assignments.

The most comprehensive assignment of Ru_3_(CO)_12_ in the 0–750 cm^–1^ region is that of Adams and Taylor^13^ and these are listed in Table 3. However, of the 42 modes in this region they only locate 26, unsurprisingly these are the infrared and Raman active modes, those that are forbidden under *D*_3h_ symmetry (*A*′_2_, *A*″_1_, *E*″) are largely unassigned.

A comparison of an experimental INS spectrum with that generated from a DFT calculation is commonly used as a means to assign the spectrum.^33,34^ We have previously employed this approach to assign the solid-state spectra of the metal hexacarbonyls, M­(CO)_6_, M = Cr, Mo, W.^31^ For these molecules the agreement between observed and calculated spectra was excellent, however, for Fe­(CO)_5_ ^30^ and Fe_2_(CO)_9_,^32^ this was not the case. As Figure 8 shows, the agreement between observed and calculated spectra is, at best, modest for Ru_3_(CO)_12_.

**Figure 8:**
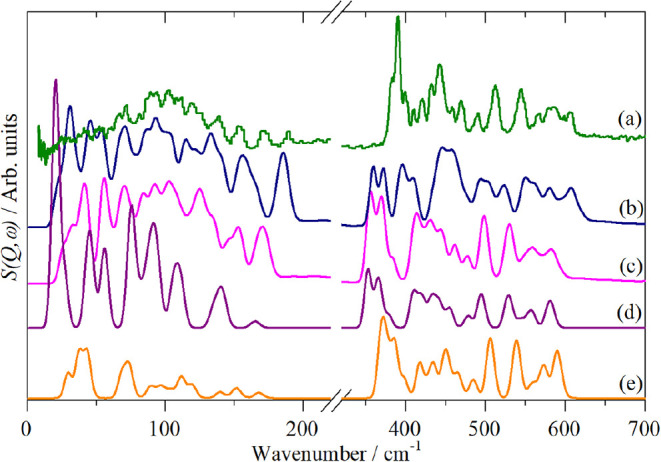
Comparison of observed and calculated (from DFT) spectra of Ru_3_(CO)_12_. (a) Experimental spectrum at 10 K, (b) DMol3 (GGA, PBE, DNP), (c) CASTEPv17 (GGA, PBE, TS, OTFG) (d) CASTEPv17 (GGA, PBE, OTFG) isolated molecule calculation with *D*_3h_ geometry imposed and (e) Gaussian09 (B3LYP, LanL2DZ). (b, c) are fully periodic calculations of the *P*2_1_/*c* structure, (d) and (e) are isolated molecule calculations with *D*_3h_ geometry imposed. See the Computational Studies part of the Experimental Section for the definition of the acronyms.

Both fully periodic calculations, (DMol^3^, Figure 8b and CASTEP, Figure 8c) and isolated molecule (Gaussian09, Figure 8d) were used. CASTEP is a periodic method that uses plane-waves, DMol^3^ and Gaussian09 use atom centered orbitals. The calculated geometry (Table 2) generally showed good agreement with the experimental data, so the discrepancies between theory and experiment are not the result of an inaccurate structure. We have previously shown^41^ that, providing the geometry was reasonably accurate (as is the case here), the mode eigenvectors that describe the motion (*i.e*. the amplitude of vibration of each atom in the mode) are relatively insensitive to the eigenvalue (transition energy). This means that the calculated transition energies can be shifted (“scaled”) to test an assignment scheme.

To simplify the problem we will initially assume *D*_3h_ symmetry in the solid state and only one molecule in the unit cell. This leads to the calculated spectrum shown in Figure 8d, the resemblance to the periodic calculation in Figure 8c is clear. Major differences occur in the 0–200 cm^–1^ region because the periodic calculation includes the 12 translational and 12 librational modes, whereas in the isolated molecule calculation only three librational modes (the intense peak at 20 cm^–1^) are present.

The calculated INS spectra do provide some useful information. By setting the transition energies for all the modes to zero, except for one irreducible representation, it is possible to display each irreducible representation in isolation. Figure 9 shows the results. It is apparent that all *A* modes (***A*** ′_**1**_, ***A*** ′_**2**_, ***A*** ″_**1**_, ***A*** ″_**2**_) have similar intensities as do *E* modes (***E*** ′, ***E*** ″) and that *E* modes are approximately twice as intense as *A* modes. This means that distinguishing *A* from *E* modes is straightforward, but that the INS does not give any information on distinguishing between the irreducible representations in each type of mode (*A* or *E*). However, by making use of the infrared and Raman selection rules, the only ambiguity is between the ***A*** ′_**2**_ and ***A*** ″_**1**_ classes.

**Figure 9:**
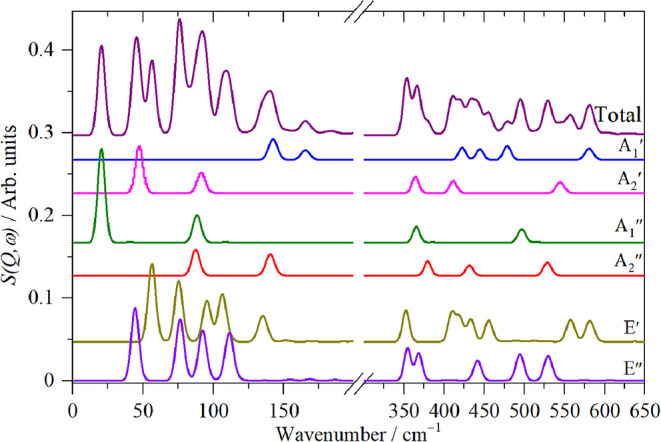
Decomposition of the DFT calculated (in *D*_3h_ symmetry) spectrum of Ru_3_(CO)_12_ into the modes of each irreducible representation.

The procedure adopted was to move each calculated mode to the nearest experimental peak. In most cases the shift required was in the range 0–20 cm^–1^. Figure 10a compares the experimental spectrum and the adjusted DFT calculation. It is apparent that the agreement in the 300–700 cm^–1^ region is excellent and very poor in the 0–200 cm^–1^ region. We will consider each region separately.

**Figure 10:**
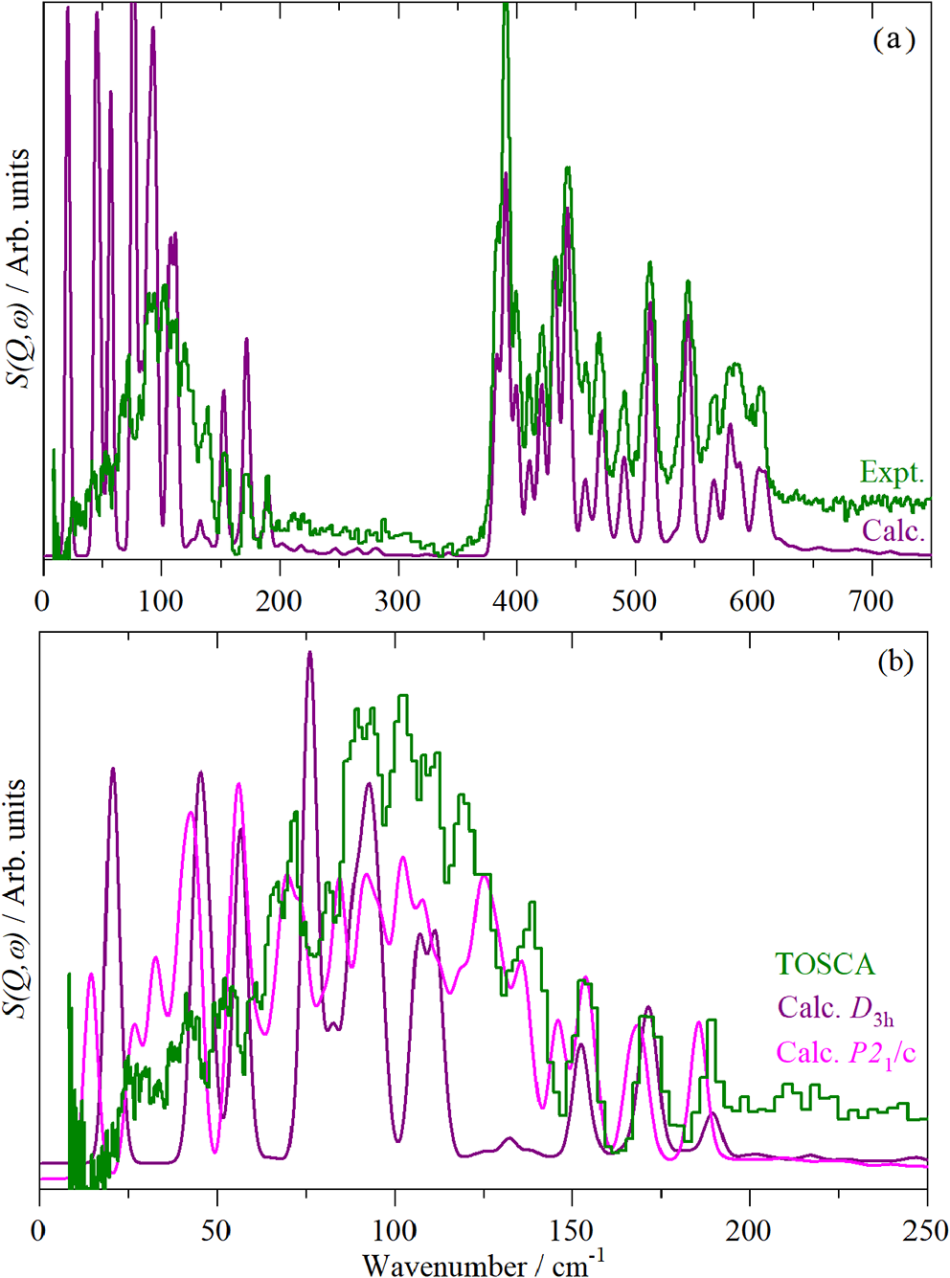
(a) Comparison of the experimental (“Expt.”) INS spectrum of Ru_3_(CO)_12_ with that calculated from an adjusted calculation of Ru_3_(CO)_12_ in *D*_3h_ symmetry (“Calc.”) and (b) as (a) but showing only the 0–250 cm^–1^ region and including the adjusted calculation of Ru_3_(CO)_12_ in the complete unit cell (“Calc. *P*2_1_/*c*”).

### The 300–700 cm^–1^ Region

It is apparent by comparison of the INS spectrum (Figure 7c) and the decomposition into the symmetry classes (Figure 9) that there must be several modes at, or near, 390 cm^–1^ in order to account for the intensity of the feature. We find five modes within 10 cm^–1^ of 390 cm^–1^ (*A*′_2_, *E*′, *A*″_1_, 2 × *E*″). Similarly for the second most intense mode at 443 cm^–1^, five modes are also required (*A*″_1_, *A*′_2_, *E*′, *A*″_2_, *E* ^″^). In both cases, with one exception, they are all δ­(Ru–C≡O) modes.

Table 3 lists the literature assignments together with our new ones. In most cases our assignments for the *A*′_1_, *A*″_2_ and *E*′ modes agree with those of Adams and Taylor.^13^ Where the disagreements arise it is because they made the assumptions that the *D*_3h_ selection rules were obeyed and that ν­(Ru–CO) modes were at lower energy than δ­(Ru–C≡O) modes. Neither of these assumptions is correct. The *C*_1_ site symmetry combined with the *C*_2h_ factor group symmetry means that *all* modes have components that are infrared and Raman active. This is particularly the case for the *E*″ modes that may have significant infrared activity, the 515 cm^–1^ mode being a case in point. The mode visualizations clearly show that both ν­(Ru–CO) and δ­(Ru–C≡O) modes are distributed across the entire range.

### The 0–250 cm^–1^ Region

The only modes that are known with any certainty^9–13^ in this region are the Ru–Ru stretch modes at 151 and 185 cm^–1^ (*E*′ and *A*′_1_ respectively). Figure 10b shows an expanded view of this region with the experimental data, the fully periodic calculation of the *P*2_1_/*c* and the *D*_3h_ isolated molecule calculation. In both calculations only the transition energies of the Ru–Ru stretch modes have been adjusted. Below 50 cm^–1^ the agreement is very poor with the modes calculated to be much stronger than observed. Adams and Taylor concluded that these were likely to be translational optic modes, a proposal that is confirmed by the mode visualizations. These are likely to show vibrational dispersion (variation of transition energy with wavevector) which is observable by INS^18^ and would have the effect of spreading the modes out. Unfortunately, given the number of atoms in the primitive cell (108), a full dispersion calculation is not feasible with our computational resources.

Above 50 cm^–1^ the *P*2_1_/*c* calculation shows surprisingly good agreement with the experimental spectrum, however, both the experimental data and the *P*2_1_/*c* calculation are in marked disagreement with the *D*_3h_ isolated molecule calculation. As both the experimental data and the *P*2_1_/*c* calculation include the librational modes, it was hoped that a comparison of the *P*2_1_/*c* and *D*_3h_ calculations would enable these to be easily pinpointed. This turns out not to be the case and the spectra are distinctly different. The most likely reason is because the librations and the δ­(OC–Ru–CO) modes have similar transition energies and belong to the same irreducible representations, they are allowed to mix. In the solid state this is likely to be case for most of the modes in the 50–150 cm^–1^ range. It also means that it is not possible to map the symmetry assignments from the isolated molecule calculation to the solid state as was done for the 300–700 cm^–1^ region. In solution, where the *D*_3h_ selection rules are valid, it is potentially possible to observe the pure modes. Unfortunately, the poor solubility of Ru_3_(CO)_12_ in virtually all solvents has so far prevented this being accomplished.

The difference between the *P*2_1_/*c* and *D*_3h_ calculations means that it is not possible to assign the modes in this region with any confidence. In Table 3, the modes in this region are as reported by DFT, adjustment would not seem to serve any purpose. Table S2 lists the DFT calculated transition energies

## Conclusions

In this work we have re-examined the structure and vibrational spectroscopy of Ru_3_(CO)_12_. The high-resolution neutron powder diffraction shows that the room temperature structure is maintained down to 10 K. The structure is highly compressible, as shown by both our variable temperature study and the ambient temperature pressure study.^7^ Our diffraction study has confirmed that in the solid state, the axial Ru–C lengths are longer than the equatorial values, Table 2.

INS spectroscopy has enabled the observation of many of the internal modes for the first time. Only some of the infrared and Raman active modes were known with any certainty, the present work has extended this to include modes that are either forbidden in *D*_3h_ symmetry or were not observed because of overlap with other modes or having intrinsically low intensity. Unfortunately, a complete and unambiguous assignment has not been possible. This is because in the 0–250 cm^–1^ region where the δ­(OC–Ru–CO) modes occur, the low crystal symmetry results in extensive mixing with the librational modes.

One of the aims of this work was to test how well DFT performed for this system. As shown in Figure 8, the answer is “poorly”. This is irrespective of whether it is a periodic calculation or an isolated molecule calculation, three different codes give similar results, all at variance with experiment. This was also found to be the case for Fe­(CO)_5_ ^30^ and Fe_2_(CO)_9_ ^32^ but not for the metal hexacarbonyls M­(CO)_6_, (M = Cr, Mo, W).^31^ In all cases the calculated geometry is in good agreement with the experimental structure.

## Experimental Section

### Materials

Triruthenium dodecacarbonyl, Ru_3_(CO)_12_ (99%), was purchased from Aldrich and used as received.

### Neutron Powder Diffraction

Ru_3_(CO)_12_ powder was transferred into an Al-alloy frame surrounding a cuboid cavity of dimensions 18 × 23 mm (*w* × *h*) perpendicular to the incident neutron beam and 15 mm depth parallel to the beam. The front and back faces of the sample were covered with 125 μm-thick vanadium-foil windows, sealed to the Al-frame with indium wire. The sample can was mounted in a closed-cycle refrigerator (CCR) mounted in the sample vacuum tank of the High-Resolution Powder Diffractometer (HRPD)^42,43^ at the ISIS Neutron and Muon Spallation Source.^44^ Temperature control was achieved by balancing the cooling from the helium exchange gas (at ∼50 mb) with direct heating of the sample holder via a cartridge heater inserted into the Al-frame. Temperatures were measured and controlled by means of a RhFe thermometer inserted in the frame on the opposite side of the sample. To ensure thermal equilibrium between the heated frame and the powder samples during a variable-temperature study requires moderately slow ramping between temperature set-points, ∼3 K min^–1^, and a wait of at least 10 min after reaching the set-point prior to commencing data acquisition, a previously established protocol.^45,46^ After an initial check at 285 K the sample temperature was reduced to 10 K and high-quality data were collected using the instrument’s 30–130 ms and 100–200 ms time-of-flight (TOF) windows. In HRPD’s highest resolution backscattering detectors (2θ = 154–176°, Δ*d*/*d* ≈ 1.0 × 10^–3^) these two windows cover *d*-spacing ranges of 0.65–2.60 Å and 2.2–4.0 Å, respectively. The two data sets were counted for 240 and 200 μA of integrated beam current, respectively. Data were then collected on warming from 25 K in 25 K increments using only the 100–200 ms TOF window, counting each for 20 μA (∼25 m of real time) for the purpose of precise lattice parameter refinement rather than structure refinement.

These data were time-focused to a common scattering angle (2θ = 168.3°), normalized to the incident spectrum and corrected for detector efficiency by reference to a V:Nb standard using the Mantid suite of neutron scattering utilities.^47,48^ Structural refinements were then carried out using the Rietveld method implemented in GSAS/Expgui^49,50^ starting from one of the previously reported structures of Ru_3_(CO)_12_.^5^ The crystal structure of Ru_3_(CO)_12_ at 10 K is provided as a supplementary Crystallographic Information File (CIFs) and has been deposited with the Cambridge Crystallographic Data Centre (deposition number 2503303). Unit-cell parameters obtained from profile refinements done with the *F*(calc) weighted method in GSAS are reported in Table S1.

### Vibrational Spectroscopy

For the INS measurements 9.6 g of Ru_3_(CO)_12_, was loaded into an In wire-sealed Al can. The sample was quenched in liquid nitrogen immediately before insertion into the indirect geometry, high resolution, broad band spectrometer TOSCA^51,52^ at ISIS^44^ and measured for 1563 μA of integrated beam current (∼8.5 h). The data were converted from time-of-flight to energy transfer using standard routines included in the Mantid suite of programs.^47,48^ (A detailed description of TOSCA and its mode of operation are given in refs 29,51 ). An empty Al can (measured at 10 K) was subtracted from the experimental spectrum.

Raman spectra were recorded with the sample in a quartz cell using a Bruker FT-Raman spectrometer (64 scans at 1 cm^–1^ resolution with 500 mW laser power at 1064 nm). Variable temperature (7–300 K) Raman spectra were recorded with a previously described,^53^ Renishaw in-Via system using 785 nm excitation.

Infrared spectra (64 scans at 4 cm^–1^ resolution with eight times zero filling (to improve the peak shape)) were recorded with a Bruker Vertex 70 FTIR spectrometer. Room temperature spectra over the range 50–4000 cm^–1^ were obtained using the Bruker Diamond ATR accessory. Spectra (300–4000 cm^–1^) between 296 and 170 K were recorded using a SpecAc Golden Gate variable temperature accessory.

### Computational Studies

DFT calculations were carried out using CASTEP^54^ (v17 and v20), DMol^3^ (version 2021)^55^ and Gaussian09.^56^ For the CASTEP and DMol^3^ calculations the initial structure was that determined at 10 K in the present work. (We note that *P*2_1_/*c* and *P*2_1_/*n* are different settings (choice of axes) within space group number 14. The crystallographic standard is to adopt the setting with the less obtuse monoclinic angle, in this case *P*2_1_/*n* as β = 100.476° in this setting versus 148.748° in *P*2_1_/*c*. The structural refinements were carried out in *P*2_1_/*n* but it was more convenient computationally to use the alternative setting of *P*2_1_/*c* and this was used for the CASTEP calculations. As the symmetry elements present and the molecular structure are the same in both settings, this has no effect on the vibrational spectroscopy. For DMol^3^ the space group was set to *P*1 because the program cannot make use of the symmetry for the vibrational calculation of a periodic system.) For the plane-wave, pseudopotential code CASTEP, exchange and correlation were approximated using the Perdew–Burke–Ernzerhof (PBE) functional,^57^ with the Tkatchenko-Scheffler (TS) dispersion correction scheme^58^ within the generalized gradient approximation (GGA). On-the-fly generated (OTFG) norm-conserving pseudopotentials were used. The plane-wave cutoff was 830 eV and the Brillouin-zone sampling of electronic states used a 6 × 4 × 4 Monkhorst–Pack grid (24 *k*-points). The equilibrium structure, an essential prerequisite for lattice dynamics calculations was obtained by Broyden-Fletcher-Goldfarb-Shanno (BFGS) geometry optimization after which the residual forces were converged to zero within |0.0072| eV Å^–1^. Phonon frequencies were obtained by diagonalization of dynamical matrices computed using density-functional perturbation theory^59^ (DFPT). An analysis of the resulting eigenvectors was used to map the computed modes to the corresponding irreducible representations of the point group and assign IUPAC symmetry labels. DFPT was also used to compute the dielectric response and the Born effective charges, and from these the mode oscillator strength tensor and infrared absorptivity were calculated. Only the Brillouin zone Γ-point (*q* = 0) modes were calculated because the low symmetry (*P*2_1_/*c*) and the large number of atoms (108) in the primitive cell made running the complete dispersion calculation impractical. For DMol^3^, the PBE functional in the GGA was used with the DNP basis set with DFT calculated semicore pseudopotentials. A 6 × 4 × 6 Monkhorst–Pack grid (72 *k*-points) was used. Vibrational transition energies were calculated by a finite displacement method. For Gaussian09 the B3LYP functional with the Los Alamos National Laboratory double-ζ basis set (LanL2DZ) basis set was used. The INS spectra were generated from the CASTEP, DMol^3^ and Gaussian09 output using the AbINS^60^ module within Mantid. AbINS also includes a parametrization of TOSCA’s resolution function and optionally allows overtones and combinations to be calculated up to the 10th order, so the calculated spectra are as close a representation of what would be measured experimentally as possible. The geometry optimized structures are provided as cif files (CASTEP and DMol^3^) or Cartesian coordinates (Gaussian) in the SI (Tables S2–S5).

## Supplementary Material



## Notes

Refined unit cell parameters as a function of temperature, structure of Ru_3_(CO)_12_ at 10 K as determined by powder neutron diffraction showing bond lengths and angles, vibrational spectra of Ru_3_(CO)_12_ in the 0–2200 cm^–1^ range, geometry optimized structures as cif files (CASTEP and DMol^3^) or Cartesian coordinates (Gaussian) (PDF)

## References

[1] Mond L., Hirtz H., Cowap M. D. (1910). Einige neue Metallkarbonyle. Z. Anorg. Chem..

[2] Manchot W., Manchot W. J. (1936). Darstellung von Rutheniumcarbonylen
und – nitrosylen. Z. Anorg. Allg. Chem..

[3] Corey E. R., Dahl E. F. (1961). Trinuclear osmium
and ruthenium carbonyls and their
identities with previously reported Os_2_(CO)_9_ and Ru_2_(CO)_9_. J. Am.
Chem. Soc..

[4] Mason R., Rae A. I. M. (1968). The crystal structure
of ruthenium carbonyl, Ru_3_(CO)_12_. J. Chem. Soc. A.

[5] Churchill M. R., Hollander F. J., Hutchinson J. P. (1977). An accurate redetermination of the
structure of triruthenium dodecacarbonyl, Ru_3_(CO)_12_. Inorg. Chem..

[6] Braga D., Grepioni F., Tedesco E., Dyson P. J., Martin C. M., Johnson B. F. G. (1995). A variable temperature
study of Ru_3_(CO)_12_ in the solid state and the
generation of alternative crystal
structures. Transition Met. Chem..

[7] Slebodnick C., Zhao J., Angel R., Hanson B. E., Song Y. Y., Liu Z., Hemley R. J. (2004). High pressure
study of Ru_3_(CO)_12_ by X-ray diffraction, Raman,
and infrared spectroscopy. Inorg. Chem..

[8] Eady C. R., Jackson P. F., Johnson B. F. G., Lewis J., Malatesta J., Mcpartlin M. C., Nelson W. J. H. (1980). Improved syntheses of the hexanuclear
clusters [Ru_6_(CO)_18_]^2–^, [HRu_6_-(CO)_18_]^−^, and H_2_Ru_6_(CO)_18_. The X-ray analysis of [HRu_6_(CO)_18_]^−^, a polynuclear carbonyl containing an
interstitial hydrogen ligand. J. Chem. Soc.,
Dalton Trans..

[9] Quicksall C. O., Spiro T. G. (1968). Raman Frequencies of Metal Cluster
Compounds: Os_3_(CO)_12_ and Ru_3_(CO)_12_. Inorg. Chem..

[10] Kettle S. F. A., Stanghellini P. L. (1979). Solid-state
Studies. 18. Low-frequency
Raman Spectra of Os_x_Ru_3‑x_(CO)_12_ (x = 0, 1, 2, 3) and of [Os_3_(CO)_12_]_
*n*
_[Ru_3_(CO)_12_]_1‑*n*
_ (0 < *n* < 1). Inorg. Chem..

[11] Battiston G. A., Bor G., Dietler U. K., Kettle S. F. A., Rossetti R., Sbrignadello G., Stanghellini P. L. (1980). Comparative infrared and Raman Spectroscopic ν­(CO)
study of Ru_3_(CO)_12_, Os_3_(CO)_12_, their mixed crystals, and the mixed triangulo cluster carbonyls
Ru_2_Os­(CO)_12_and RuOs_2_(CO)_12_. Inorg. Chem..

[12] Kishner S., Fitzatrick P. J., Plowman K. R., Butler I. S. (1981). Variable-temperature
(295—15 K) Raman spectra of dodecacarbonyl-triangulo-ruthenium­(O)
and -osmium­(O), M_3_(CO)_12_ (M = Ru, Os). J. Mol. Struct..

[13] Adams D. M., Taylor I. D. (1982). Solid-state metal carbonyls. Part
4.-An infrared and
far-infrared study of M_3_(CO)_12_, M = Ru, Os. J. Chem. Soc., Faraday Trans. 2.

[14] Gilson T. R. (1984). Vibrational
studies of the cluster carbonyls of ruthenium and osmium. Part 1.
Raman solution data for [Ru_3_(CO)_12_], [Os_3_(CO)_12_] and [Ru_4_H_4_(CO)_12_] in the CO stretching region. J. Chem.
Soc., Dalton Trans..

[15] Gilson T. R., Evans J. (1984). Vibrational studies
of the cluster carbonyls of ruthenium and osmium.
Part 2. Single-crystal Raman and infrared data for [Ru_3_(CO)_12_] in the CO stretching region. J. Chem. Soc., Dalton Trans..

[16] Butler I. S., Xu Z. H., Darensbourg D. J., Pala M. (1987). FT-IR, photoacoustic
and micro-Raman spectra of the dodecacarbonyltriruthenium (O) complexes
Ru_3_(^13^CO)_12_ and Ru_3_(CO)_12_. J. Raman Spectrosc..

[17] John A., DiBenedetto J. A., Ryba D. W., Ford P. C. (1989). Reaction dynamics
of photosubstitution intermediates of the triruthenium cluster Ru_3_(CO)_12_ as studied by flash photolysis with infrared
detection. Inorg. Chem..

[18] Kong Q., Lee J. H., Plech A., Wulff M., Ihee H., Koch M. H. J. (2008). Ultrafast X-ray
solution scattering reveals an unknown
reaction intermediate in the photolysis of Ru_3_(CO)_12_. Angew. Chem., Int. Ed..

[19] Dong X., Yang F., Zhao J., Wang J. (2018). Efficient
intramolecular
vibrational excitonic energy transfer in Ru_3_(CO)_12_ cluster revealed by two-dimensional infrared spectroscopy. J. Phys. Chem. B.

[20] Gross D. C., Ford P. C. (1982). Kinetics of carbon monoxide activation:
reactions of
methoxide and of hydroxide with ruthenium and iron carbonyls. Inorg. Chem..

[21] Reeves J. T., Tan Z., Marsini M. A., Han Z. S., Xu Y., Reeves D. C., Lee H., Lu B. Z., Senanayake C. H. (2013). A practical procedure for reduction
of primary, secondary and tertiary amides to amines. Adv. Synth. Catal..

[22] Murray R. E., Walter E. L., Doll K. M. (2014). Tandem isomerization-decarboxylation
for converting alkenoic fatty acids into alkenes. ACS Catal..

[23] Wenchao
Zhai W., Li B., Wang B. (2018). catalyzed dehydrogenative S–N
coupling of indoles with hydrosilanes without additive. Tetrahedron.

[24] Eliseev O. L., Bondarenko T. N., Churikova A. D., Lapidus A. L. (2022). Ruthenium-catalyzed
methoxycarbonylation of styrene. Mendeleev Commun..

[25] Bruce L. A., Hoang M., Hughes A. E., Turney T. W. (1993). Ruthenium promotion
of Fischer–Tropsch synthesis over coprecipitated cobalt/ceria
catalysts. Appl. Catal., A.

[26] Kim T. W., Oh J., Suh Y.-W. (2017). Hydrogenation
of 2-benzylpyridine over alumina-supported
Ru catalysts: Use of Ru_3_(CO)_12_ as a Ru precursor. Appl. Catal., A.

[27] Ji S., Chen Y., Fu Q., Chen Y., Dong J., Chen W., Li Z., Wang Y., Gu L., He W., Chen C., Peng Q., Huang Y., Duan X., Wang D., Draxl C., Li Y. (2017). Confined pyrolysis
within metal–organic frameworks to form uniform ru3 clusters
for efficient oxidation of alcohols. J. Am.
Chem. Soc..

[28] Kisi, E. H. ; Howard, C. J. Applications of Neutron Powder Diffraction; Oxford University Press, 2008.

[29] Mitchell, P. C. H. ; Parker, S. F. ; Ramirez-Cuesta, A. J. ; Tomkinson, J. Vibrational Spectroscopy with Neutrons, with Applications in Chemistry, Biology, Materials Science and Catalysis; World Scientific, Singapore, 2005.

[30] Fortes A. D., Parker S. F. (2022). Structure and spectroscopy of iron
pentacarbonyl, Fe­(CO)_5_. J. Am. Chem.
Soc..

[31] Parker S. F., Jayasooriya U. A. (2019). Assignment of the solid state spectra of the Group
VI hexacarbonyls by inelastic neutron scattering spectroscopy. Phys. Chem. Chem. Phys..

[32] Parker S.
F. (2022). Assignment
of the vibrational spectra of diiron nonacarbonyl, Fe_2_(CO)_9_. Physchem.

[33] Armstrong J., O’Malley A. J., Ryder M. R., Butler K. T. (2020). Understanding dynamic
properties of materials using neutron spectroscopy and atomistic simulation. J. Phys. Commun..

[34] Nolasco M. M., Vaz P. D., Serrano R. A. F., Martins J. T., Araújo C. F., Ribeiro-Claro P. (2025). Computational spectroscopy for crystalline
materials:
from structure to properties. CrystEngComm.

[35] Cochran, W. The Dynamics of Atoms in Crystals; Arnold, London, 1973.

[36] Hazen R. M., Downs R. T., Prewitt C. T. (2000). Principles of comparative crystal
chemistry. Rev. Mineral. Geochem..

[37] Abdi, H. The Eigen Decomposition; Eigenvalues and Eigenvectors. In Encylopedia of Measurements and Statistics; Salkind, N. J. , Ed.; Sage Publications: Thousand Oaks, CA, 2007.

[38] Hashash Y.
M. A., Yao J. I.–C., Wotring D. C. (2003). Glyph and hyperstreamline
representation of stress and strain tensors and material constitutive
response. Int. J. Numer. Anal. Methods Geomech..

[39] Kaminski, W. WinTensor 1.1 2004 http://cad4.cpac.washington.edu/WinTensorhome/WinTensor.htm.

[40] Fateley, W. G. ; Dollish, F. R. ; McDevitt, N. T. ; Bentley, F. E. Infrared and Raman Selection Rules for Molecular and Lattice Vibrations: The Correlation Method; Wiley-Interscience, New York, 1972.

[41] Tomkinson J., Parker S. F. (2011). Exploiting the quasi-invariance of
molecular vibrational
eigenvectors. Spectrochim. Acta, Part A.

[42] Ibberson, R. M. ; David, W. I. F. ; Knight, K. S. The High Resolution Neutron Powder Diffractometer (HRPD) at ISIS – A User Guide, RAL-92-031; Rutherford Appleton Laboratory: U.K., 1992, http://purl.org/net/epubs/work/25156.

[43] Ibberson R. M. (2009). Design
and performance of the new supermirror guide on HRPD at ISIS. Nucl. Instrum. Methods Phys. Res., Sect. A.

[44] https://www.isis.stfc.ac.uk/Pages/About.aspx.

[45] Fortes A. D. (2018). Accurate
and precise lattice parameters of H_2_O and D_2_O ice I*h* between 1.6 and 270 K from high-resolution
time-of-flight neutron powder diffraction data. Acta Crystallogr., Sect. B: Struct. Sci., Cryst. Eng. Mater..

[46] Fortes A. D., Capelli S. C. (2018). H/D isotope effect
on the molar volume and thermal
expansion of benzene. Phys. Chem. Chem. Phys..

[47] Arnold O., Bilheux J., Borreguero J. (2014). Mantid—Data analysis
and visualization package for neutron scattering and μSR experiments. Nucl. Instrum. Methods Phys. Res., Sect. A.

[48] MANTID . Manipulation and Analysis Toolkit for Instrument Data Mantid Project 2013 10.5286/SOFTWARE/MANTID.

[49] Larson, A. C. ; Von Dreele, R. B. General Structure Analysis System (GSAS); Los Alamos National Laboratory Report, LAUR, 1994; pp 86–748.

[50] Toby B. H. (2001). *EXPGUI*, a graphical user interface for *GSAS*. J. Appl. Crystallogr..

[51] Parker S. F., Fernandez-Alonso F., Ramirez-Cuesta A. J., Tomkinson J., Rudić S., Pinna R. S., Gorini G., Fernández
Castañon J. (2014). Recent and future developments on TOSCA at ISIS. J. Phys.: Conf. Ser..

[52] Pinna R. S., Rudić S., Parker S. F., Armstrong J., Zanetti M., Škoro G., Waller S. P., Zacek D., Smith C. A., Capstick M. J., McPhail D. J., Pooley D. E., Howells G. D., Gorini G., Fernandez-Alonso F. (2018). The neutron
guide upgrade of the TOSCA spectrometer. Nucl.
Instrum. Methods Phys. Res., Sect. A.

[53] Adams M. A., Parker S. F., Fernandez-Alonso F., Cutler D. J., Hodges C., King A. (2009). Simultaneous neutron
scattering and Raman scattering. Appl. Spectrosc..

[54] Clark S. J., Segall M. D., Pickard C. J., Hasnip P. J., Probert M. J., Refson K., Payne M. C. (2005). First principles
methods using CASTEP. Z. Kristallogr. - Cryst.
Mater..

[55] Delley B. (2000). From molecules
to solids with the DMol^3^ approach. J. Chem. Phys..

[56] Frisch, M. ; Trucks, G. W. ; Schlegel, H. B. ; Scuseria, G. E. ; Robb, M. A. ; Cheeseman, J. R. ; Scalmani, G. ; Barone, V. ; Petersson, G. A. ; Nakatsuji, H. ; Li, X. ; Caricato, M. ; Marenich, A. ; Bloino, J. ; Janesko, B. G. ; Gomperts, R. ; Mennucci, B. ; Hratchian, H. P. ; Ortiz, J. V. ; Izmaylov, A. F. ; Sonnenberg, J. L. ; Williams-Young, D. ; Ding, F. ; Lipparini, F. ; Egidi, F. ; Goings, J. ; Peng, B. ; Petrone, A. ; Henderson, T. ; Ranasinghe, D. ; Zakrzewski, V. G. ; Gao, J. ; Rega, N. ; Zheng, G. ; Liang, W. ; Hada, M. ; Ehara, M. ; Toyota, K. ; Fukuda, R. ; Hasegawa, J. ; Ishida, M. ; Nakajima, T. ; Honda, Y. ; Kitao, O. ; Nakai, H. ; Vreven, T. ; Throssell, K. ; Peralta, J. E. ; Ogliaro, F. ; Bearpark, M. ; Heyd, J. J. ; Brothers, E. ; Kudin, K. N. ; Staroverov, V. N. ; Keith, T. ; Kobayashi, R. ; Normand, J. ; Raghavachari, K. ; Rendell, A. ; Burant, J. C. ; Iyengar, S. S. ; Tomasi, J. ; Cossi, M. ; Millam, J. M. ; Klene, M. ; Adamo, C. ; Cammi, R. ; Ochterski, J. W. ; Martin, R. L. ; Morokuma, K. ; Farkas, O. ; Foresman, J. B. ; Fox, D. J. Gaussian 09; Gaussian, Inc.: Wallingford CT, 2016.

[57] Perdew J. P., Burke K., Ernzerhof M. (1996). Generalized
gradient approximation
made simple. Phys. Rev. Lett..

[58] Tkatchenko A., Scheffler M. (2009). Accurate molecular
van der Waals interactions from
ground-state electron density and free-atom reference data. Phys. Rev. Lett..

[59] Refson K., Clark S. J., Tulip P. R. (2006). Variational
density-functional perturbation
theory for dielectrics and lattice dynamics. Phys. Rev. B.

[60] Dymkowski K., Parker S. F., Fernandez-Alonso F., Mukhopadhyay S. (2018). AbINS: the
modern software for INS interpretation. Phys.
B.

[61] Parker, S. F. ; Fortes, A. D. STFC ISIS Facility. 2020 10.5286/ISIS.E.RB2010031. https://doi.org/10.5286/ISIS.E.RB2010031.

[62] Parker, S. F. ; Fortes, A. D. STFC ISIS Facility. 2020 10.5286/ISIS.E.RB2010040. https://doi.org/10.5286/ISIS.E.RB2010040.

