# Supplementary material for: Structure and Spectroscopy of Triruthenium Dodecacarbonyl, Ru3(CO)12
Source: Inorg Chem. 2026 Jan 5;65(2):1677–87. doi: 10.1021/acs.inorgchem.5c05397 (PMC12820925; doi:10.1021/acs.inorgchem.5c05397)
Supplement: Supplementary file 1 [file ic5c05397_si_001.pdf]

Supporting Information for:

Structure and spectroscopy of triruthenium dodecacarbonyl, $Ru_3(CO)_{12}$

Stewart F. Parker*[a] and A. Dominic Fortes[a]

[a]ISIS Neutron and Muon Facility, STFC Rutherford Appleton Laboratory, Harwell Science and Innovation Campus, Chilton, Oxfordshire, OX11 0QX, UK.

E-mail: stewart.parker@stfc.ac.uk

Table of Contents

Table S1. Refined unit cell parameters of $Ru_3(CO)_{12}$ as a function of temperature. **S2**

Figure S1. Structure of $Ru_3(CO)_{12}$ at 10 K as determined by powder neutron diffraction. **S3**

Figure S2. Vibrational spectra of $Ru_3(CO)_{12}$. **S5**

Table S2. Crystallographic information file (cif) of the CASTEP geometry optimised structure of $Ru_3(CO)_{12}$ in $P2_1/c$ **S6**

Table S3. Crystallographic information file (cif) of the CASTEP geometry optimised structure of $Ru_3(CO)_{12}$ with D_{3h} symmetry imposed. **S8**

Table S4. Crystallographic information file (cif) of the DMol^3 geometry optimised structure of $Ru_3(CO)_{12}$ in $P1$. **S10**

Table S5. Cartesian coordinates of the Gaussian geometry optimised structure of $Ru_3(CO)_{12}$ with D_{3h} symmetry imposed. **S16**

Table S1. Refined unit cell parameters of $Ru_3(CO)_{12}$ as a function of temperature. Numbers in parentheses report the estimated standard uncertainty in the last quoted digit. These values are plotted in Figure 1 and are further analysed in the main text.

T (K)	*a* (Å)	*b* (Å)	*c* (Å)	β (°)	V (Å^3)
300	8.11776(7)	14.86572(9)	14.61545(9)	100.652(1)	1733.35(2)
275	8.09928(6)	14.83213(8)	14.58316(9)	100.628(1)	1721.82(1)
250	8.08190(6)	14.80045(8)	14.55228(9)	100.605(1)	1710.95(1)
225	8.06472(6)	14.77094(8)	14.52284(9)	100.585(1)	1700.57(1)
200	8.04816(7)	14.74286(8)	14.49448(9)	100.566(1)	1690.65(1)
175	8.03218(6)	14.71661(8)	14.46691(8)	100.551(1)	1681.17(1)
150	8.01695(6)	14.69212(8)	14.44090(8)	100.535(1)	1672.27(1)
125	8.00205(7)	14.66875(9)	14.41590(9)	100.522(1)	1663.69(2)
100	7.98793(6)	14.64773(8)	14.39214(9)	100.509(1)	1655.71(1)
75	7.97527(7)	14.62880(9)	14.37091(9)	100.498(1)	1648.57(2)
50	7.96414(6)	14.61355(8)	14.35271(8)	100.486(1)	1642.53(1)
25	7.95651(6)	14.60289(7)	14.33975(8)	100.477(1)	1638.33(1)
10	7.95469(3)	14.60019(4)	14.33637(4)	100.4749(3)	1637.277(7)

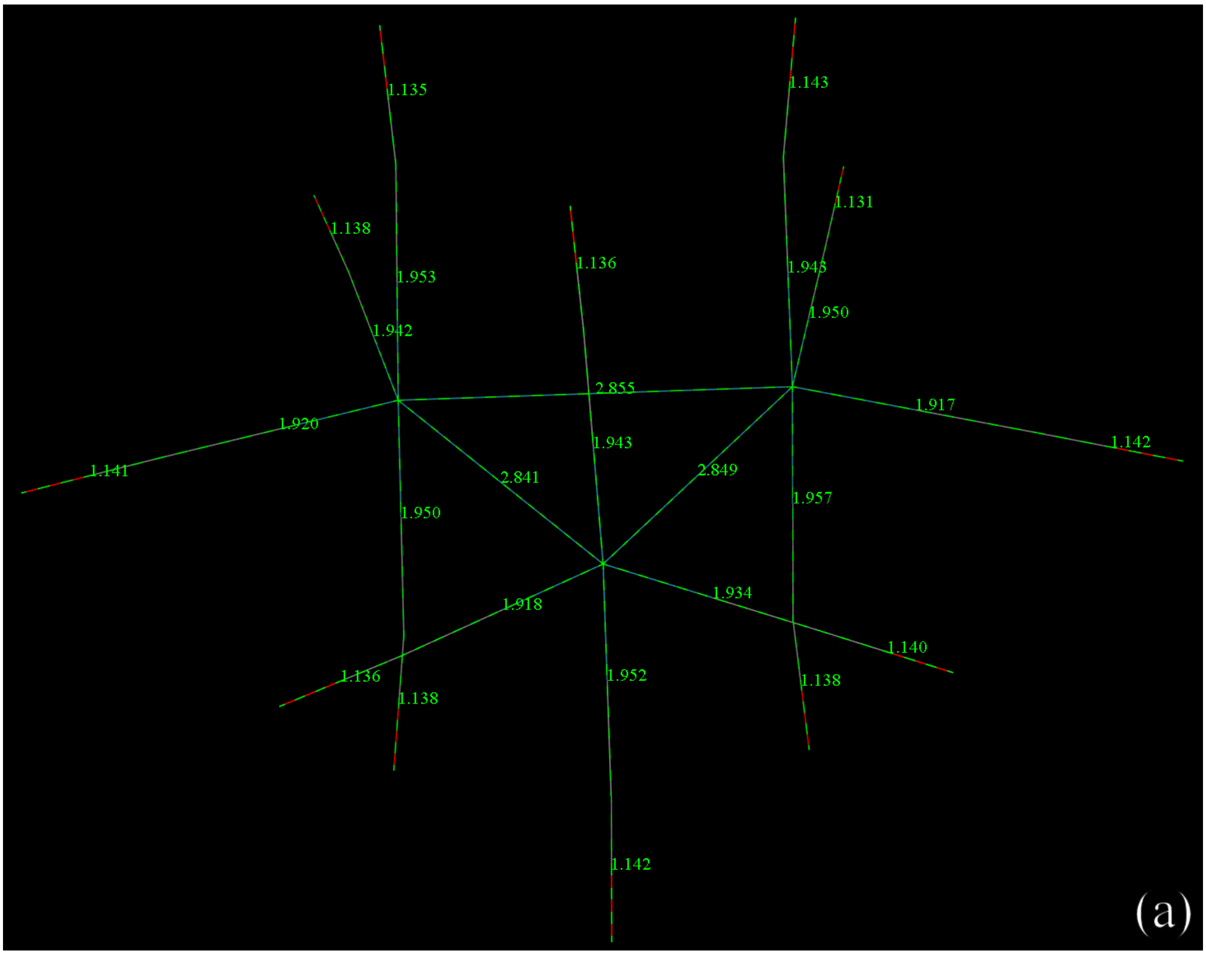

1.135
1.143
1.131
1.138
1.136
1.953
1.943
1.950
1.942
2.855
1.917
1.920
1.943
1.142
1.141
2.841
2.849
1.957
1.950
1.918
1.934
1.140
1.136
1.952
1.138
1.138
1.142
(a)

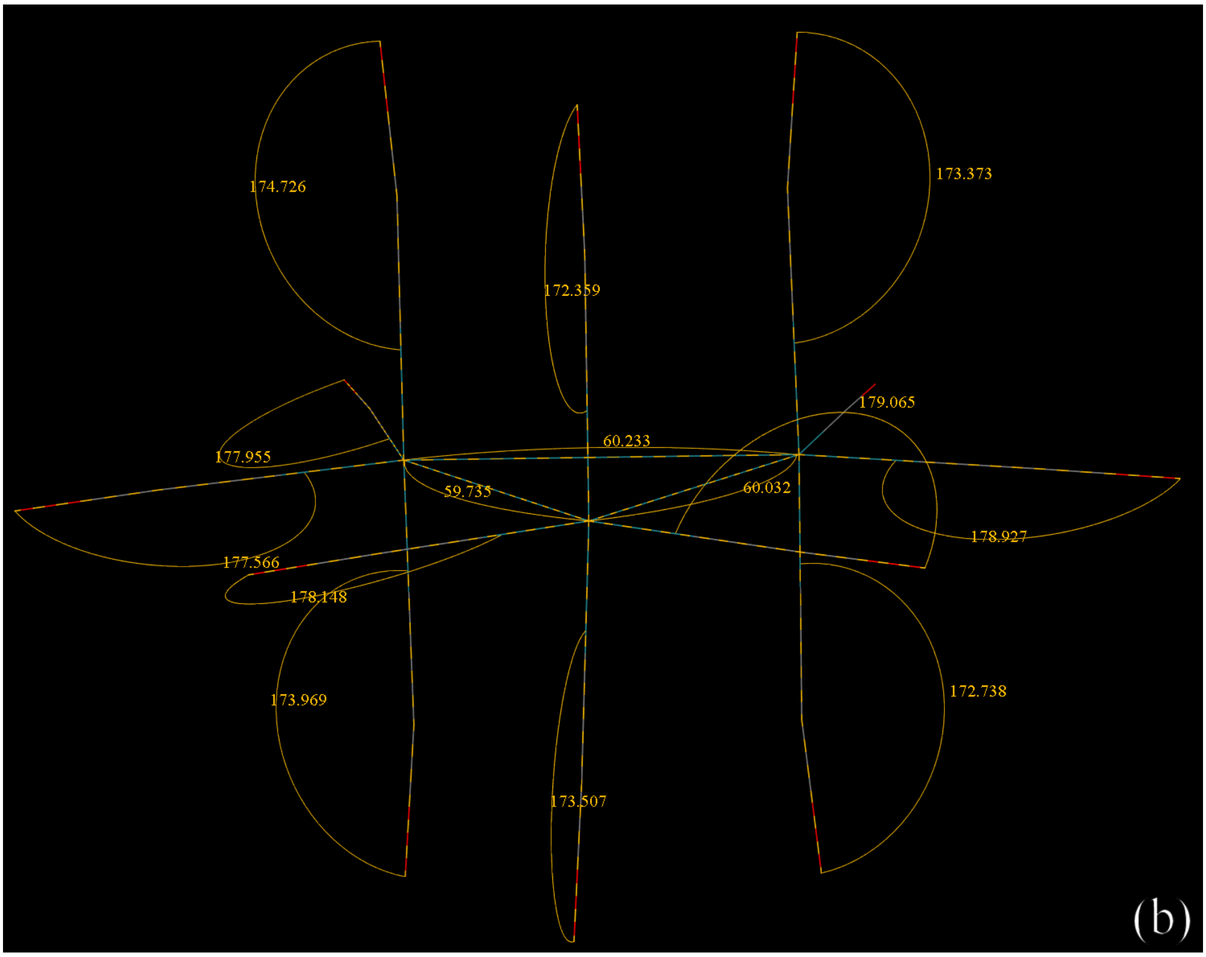

Figure S1. Structure of $Ru_3(CO)_{12}$ at 10 K as determined by powder neutron diffraction: (a) bond lengths (Å) and (b) bond angles (°).

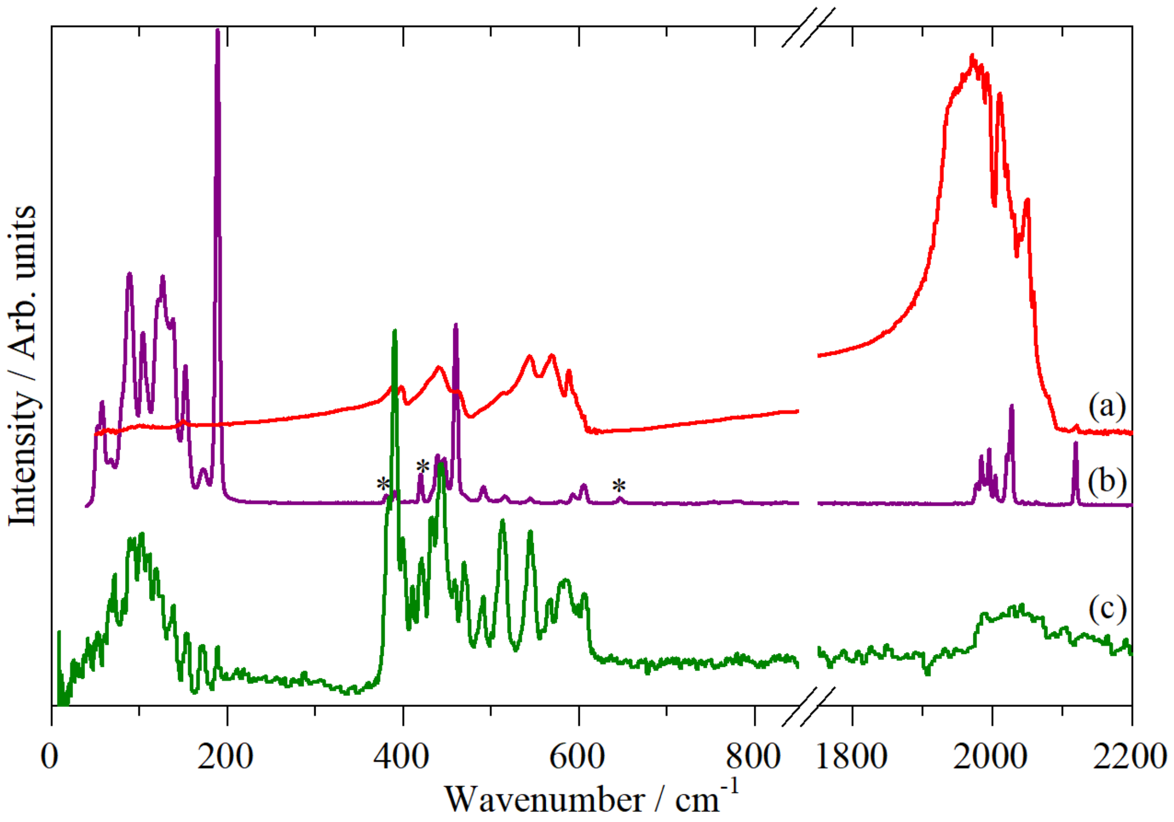

Figure S2. Vibrational spectra of $Ru_3(CO)_{12}$. (a) Infrared at 296 K, (b) Raman (785 nm) at 7 K and (c) INS at 10 K. In (b) the asterisks denote bands due to the sapphire window of the sample holder.

Table S2. Crystallographic information file (cif) of the CASTEP geometry optimised structure of $Ru_3(CO)_{12}$ in $P2_1/c$.

```
data_Ru3CO12_P21-c
_audit_creation_date              2025-10-29
_audit_creation_method            'Materials Studio'
_symmetry_space_group_name_H-M    'P21/C'
_symmetry_Int_Tables_number       14
_symmetry_cell_setting            monoclinic
loop_
_symmetry_equiv_pos_as_xyz
  x,y,z
  -x,y+1/2,-z+1/2
  -x,-y,-z
  x,-y+1/2,z+1/2
_cell_length_a                    14.5590
_cell_length_b                    14.7980
_cell_length_c                    15.2957
_cell_angle_alpha                 90.0000
_cell_angle_beta                  148.7004
_cell_angle_gamma                 90.0000
loop_
_atom_site_label
_atom_site_type_symbol
_atom_site_fract_x
_atom_site_fract_y
_atom_site_fract_z
_atom_site_U_iso_or_equiv
_atom_site_adp_type
_atom_site_occupancy
Ru1    Ru   -0.21841  -0.02670  -0.43924   0.00036  Uiso   1.00
Ru2    Ru   -0.23381   0.17120  -0.43971   0.00037  Uiso   1.00
Ru3    Ru   -0.35838   0.07950  -0.69911   0.00035  Uiso   1.00
C1     C    -0.45985  -0.03185  -0.58508   0.00048  Uiso   1.00
C2     C     0.01898  -0.01532  -0.29874   0.00060  Uiso   1.00
C3     C    -0.25083  -0.14397  -0.52073   0.00044  Uiso   1.00
C4     C    -0.11233  -0.05247  -0.23862   0.00056  Uiso   1.00
C5     C    -0.47777   0.16222  -0.59076   0.00044  Uiso   1.00
C6     C     0.00619   0.17694  -0.29523   0.00056  Uiso   1.00
C7     C    -0.13705   0.18586  -0.24314   0.00061  Uiso   1.00
C8     C    -0.28087   0.29248  -0.52059   0.00049  Uiso   1.00
C9     C    -0.59824   0.07136  -0.84244   0.00037  Uiso   1.00
C10    C    -0.11752   0.09584  -0.55073   0.00039  Uiso   1.00
C11    C    -0.43194   0.18528  -0.82490   0.00056  Uiso   1.00
C12    C    -0.40461  -0.02394  -0.81311   0.00037  Uiso   1.00
O1     O    -0.59336  -0.04473  -0.66091   0.00066  Uiso   1.00
O2     O     0.16068  -0.01975  -0.20961   0.00075  Uiso   1.00
O3     O    -0.27015  -0.21249  -0.56984   0.00065  Uiso   1.00
O4     O    -0.04729  -0.06836  -0.12027   0.00105  Uiso   1.00
O5     O    -0.61420   0.16723  -0.67024   0.00082  Uiso   1.00
```

O6 O 0.14630 0.19117 -0.20583 0.00077 Uiso 1.00
O7 O -0.08041 0.19672 -0.12870 0.00106 Uiso 1.00
O8 O -0.30690 0.36322 -0.56919 0.00079 Uiso 1.00
O9 O -0.74410 0.06927 -0.94176 0.00067 Uiso 1.00
O10 O 0.01086 0.10832 -0.48401 0.00062 Uiso 1.00
O11 O -0.47232 0.24848 -0.89564 0.00067 Uiso 1.00
O12 O -0.43167 -0.08455 -0.87941 0.00062 Uiso 1.00
loop_
_geom_bond_atom_site_label_1
_geom_bond_atom_site_label_2
_geom_bond_distance
_geom_bond_site_symmetry_2
_ccdc_geom_bond_type
Ru1 Ru2 2.937 . S
Ru1 Ru3 2.929 . S
Ru1 C1 1.984 . S
Ru1 C2 1.975 . S
Ru1 C3 1.945 . S
Ru1 C4 1.962 . S
Ru1 O3 3.090 . S
Ru2 Ru3 2.929 . S
Ru2 C5 1.987 . S
Ru2 C6 1.976 . S
Ru2 C7 1.958 . S
Ru2 C8 1.942 . S
Ru2 O8 3.087 . S
Ru3 C9 1.983 . S
Ru3 C10 1.976 . S
Ru3 C11 1.944 . S
Ru3 C12 1.957 . S
Ru3 O12 3.100 . S
C1 O1 1.143 . S
C2 O2 1.146 . S
C3 O3 1.145 . D
C4 O4 1.140 . D
C5 O5 1.141 . S
C6 O6 1.144 . S
C7 O7 1.141 . D
C8 O8 1.145 . D
C9 O9 1.142 . S
C10 O10 1.144 . S
C11 O11 1.142 . D
C12 O12 1.142 . D

Table S3. Crystallographic information file (cif) of the CASTEP geometry optimised structure of $Ru_3(CO)_{12}$ with D_{3h} symmetry imposed.

```
data_Ru3CO12_P-62m
_audit_creation_date              2025-10-29
_audit_creation_method            'Materials Studio'
_symmetry_space_group_name_H-M    'P-62M'
_symmetry_Int_Tables_number       189
_symmetry_cell_setting            hexagonal
loop_
_symmetry_equiv_pos_as_xyz
  x,y,z
  -y,x-y,z
  -x+y,-x,z
  x,y,-z
  -y,x-y,-z
  -x+y,-x,-z
  y,x,-z
  x-y,-y,-z
  -x,-x+y,-z
  y,x,z
  x-y,-y,z
  -x,-x+y,z
_cell_length_a                    15.0000
_cell_length_b                    15.0000
_cell_length_c                    15.0000
_cell_angle_alpha                 90.0000
_cell_angle_beta                  90.0000
_cell_angle_gamma                 120.0000
loop_
_atom_site_label
_atom_site_type_symbol
_atom_site_fract_x
_atom_site_fract_y
_atom_site_fract_z
_atom_site_U_iso_or_equiv
_atom_site_adp_type
_atom_site_occupancy
C1     C     1.11119  -0.00000   0.63226   0.00048  Uiso   1.00
O1     O     1.12215  -0.00000   0.70770   0.00066  Uiso   1.00
Ru1    Ru    1.11326   0.00000   0.50000   0.00036  Uiso   1.00
C3     C     1.13415  -0.11858   0.50000   0.00044  Uiso   1.00
O3     O     1.14791  -0.18700   0.50000   0.00065  Uiso   1.00
loop_
_geom_bond_atom_site_label_1
_geom_bond_atom_site_label_2
_geom_bond_distance
_geom_bond_site_symmetry_2
_ccdc_geom_bond_type
C1     Ru1     1.984   .     S
```

C1	O1	1.143	.	S
Ru1	Ru1	2.943	2_645	S
Ru1	Ru1	2.943	3_765	S
Ru1	C1	1.984	4_556	S
Ru1	C3	1.954	.	S
Ru1	C3	1.954	8_556	S
Ru1	O3	3.098	.	S
Ru1	O3	3.098	8_556	S
C3	O3	1.143	.	D

Table S4. Crystallographic information file (cif) of the DMol^3 geometry optimised structure of $Ru_3(CO)_{12}$ in *P*1.

```
data_Ru3CO12_P21-c_DMol
_audit_creation_date              2025-10-29
_audit_creation_method            'Materials Studio'
_symmetry_space_group_name_H-M    'P1'
_symmetry_Int_Tables_number       1
_symmetry_cell_setting            triclinic
loop_
_symmetry_equiv_pos_as_xyz
  x,y,z
_cell_length_a                    14.2850
_cell_length_b                    14.5400
_cell_length_c                    15.0048
_cell_angle_alpha                 90.0000
_cell_angle_beta                  148.7104
_cell_angle_gamma                 90.0000
loop_
_atom_site_label
_atom_site_type_symbol
_atom_site_fract_x
_atom_site_fract_y
_atom_site_fract_z
_atom_site_U_iso_or_equiv
_atom_site_adp_type
_atom_site_occupancy
Ru1    Ru    0.78160  -0.02642   1.06044   0.00036  Uiso   1.00
Ru2    Ru    0.76473   0.17182   1.05969   0.00037  Uiso   1.00
Ru3    Ru    0.64161   0.08010   0.80014   0.00035  Uiso   1.00
C4     C     0.53873  -0.03183   0.91279   0.00048  Uiso   1.00
C5     C     1.01907  -0.01604   1.19969   0.00060  Uiso   1.00
C6     C     0.74928  -0.14415   0.98007   0.00044  Uiso   1.00
C7     C     0.88832  -0.05200   1.26176   0.00056  Uiso   1.00
C8     C     0.51987   0.16206   0.90799   0.00044  Uiso   1.00
C9     C     1.00709   0.17620   1.20750   0.00056  Uiso   1.00
C10    C     0.86066   0.18743   1.25589   0.00061  Uiso   1.00
C11    C     0.71848   0.29318   0.97954   0.00049  Uiso   1.00
C12    C     0.40109   0.07094   0.65659   0.00037  Uiso   1.00
C13    C     0.88412   0.09677   0.95093   0.00039  Uiso   1.00
C14    C     0.56755   0.18548   0.67342   0.00056  Uiso   1.00
C15    C     0.59581  -0.02315   0.68611   0.00037  Uiso   1.00
O16    O     0.40077  -0.04389   0.83436   0.00066  Uiso   1.00
O17    O     1.16586  -0.01834   1.29300   0.00075  Uiso   1.00
O18    O     0.72997  -0.21543   0.93126   0.00065  Uiso   1.00
O19    O     0.95659  -0.06847   1.38459   0.00105  Uiso   1.00
O20    O     0.37629   0.16592   0.82075   0.00082  Uiso   1.00
O21    O     1.15073   0.18993   1.29762   0.00077  Uiso   1.00
O22    O     0.92105   0.19751   1.37532   0.00106  Uiso   1.00
O23    O     0.69263   0.36667   0.93162   0.00079  Uiso   1.00
```

O24	O	0.25254	0.07095	0.55871	0.00067	Uiso	1.00
O25	O	1.01798	0.10907	1.02213	0.00062	Uiso	1.00
O26	O	0.52546	0.25033	0.59971	0.00067	Uiso	1.00
O27	O	0.56844	-0.08577	0.61840	0.00062	Uiso	1.00
Ru28	Ru	0.21842	0.47358	0.43958	0.00036	Uiso	1.00
Ru29	Ru	0.23527	0.67182	0.44031	0.00037	Uiso	1.00
Ru30	Ru	0.35839	0.58010	0.69986	0.00035	Uiso	1.00
C31	C	0.46128	0.46818	0.58720	0.00048	Uiso	1.00
C32	C	-0.01903	0.48394	0.30035	0.00060	Uiso	1.00
C33	C	0.25066	0.35585	0.51989	0.00044	Uiso	1.00
C34	C	0.11170	0.44800	0.23825	0.00056	Uiso	1.00
C35	C	0.48013	0.66206	0.59201	0.00044	Uiso	1.00
C36	C	-0.00709	0.67620	0.29250	0.00056	Uiso	1.00
C37	C	0.13934	0.68743	0.24411	0.00061	Uiso	1.00
C38	C	0.28152	0.79318	0.52046	0.00049	Uiso	1.00
C39	C	0.59891	0.57094	0.84341	0.00037	Uiso	1.00
C40	C	0.11588	0.59677	0.54907	0.00039	Uiso	1.00
C41	C	0.43245	0.68548	0.82658	0.00056	Uiso	1.00
C42	C	0.40419	0.47685	0.81389	0.00037	Uiso	1.00
O43	O	0.59925	0.45613	0.66562	0.00066	Uiso	1.00
O44	O	-0.16582	0.48163	0.20705	0.00075	Uiso	1.00
O45	O	0.26991	0.28458	0.56870	0.00065	Uiso	1.00
O46	O	0.04344	0.43154	0.11542	0.00105	Uiso	1.00
O47	O	0.62371	0.66592	0.67925	0.00082	Uiso	1.00
O48	O	-0.15073	0.68993	0.20238	0.00077	Uiso	1.00
O49	O	0.07895	0.69751	0.12468	0.00106	Uiso	1.00
O50	O	0.30737	0.86667	0.56838	0.00079	Uiso	1.00
O51	O	0.74746	0.57095	0.94129	0.00067	Uiso	1.00
O52	O	-0.01798	0.60907	0.47787	0.00062	Uiso	1.00
O53	O	0.47454	0.75033	0.90029	0.00067	Uiso	1.00
O54	O	0.43156	0.41423	0.88160	0.00062	Uiso	1.00
Ru55	Ru	0.21840	1.02642	-0.06044	0.00036	Uiso	1.00
Ru56	Ru	0.23527	0.82818	-0.05969	0.00037	Uiso	1.00
Ru57	Ru	0.35839	0.91990	0.19986	0.00035	Uiso	1.00
C58	C	0.46127	1.03183	0.08721	0.00048	Uiso	1.00
C59	C	-0.01907	1.01604	-0.19969	0.00060	Uiso	1.00
C60	C	0.25072	1.14415	0.01993	0.00044	Uiso	1.00
C61	C	0.11168	1.05200	-0.26176	0.00056	Uiso	1.00
C62	C	0.48013	0.83794	0.09201	0.00044	Uiso	1.00
C63	C	-0.00709	0.82380	-0.20750	0.00056	Uiso	1.00
C64	C	0.13934	0.81257	-0.25589	0.00061	Uiso	1.00
C65	C	0.28152	0.70682	0.02046	0.00049	Uiso	1.00
C66	C	0.59891	0.92906	0.34341	0.00037	Uiso	1.00
C67	C	0.11588	0.90323	0.04907	0.00039	Uiso	1.00
C68	C	0.43245	0.81452	0.32658	0.00056	Uiso	1.00
C69	C	0.40419	1.02315	0.31389	0.00037	Uiso	1.00
O70	O	0.60434	1.03627	0.17343	0.00066	Uiso	1.00
O71	O	-0.16586	1.01834	-0.29300	0.00075	Uiso	1.00
O72	O	0.27003	1.21543	0.06874	0.00065	Uiso	1.00
O73	O	0.04341	1.06847	-0.38459	0.00105	Uiso	1.00

```
O74    O     0.62371   0.83408   0.17925   0.00082  Uiso   1.00
O75    O    -0.15073   0.81007  -0.29762   0.00077  Uiso   1.00
O76    O     0.07895   0.80249  -0.37532   0.00106  Uiso   1.00
O77    O     0.30737   0.63333   0.06838   0.00079  Uiso   1.00
O78    O     0.74746   0.92905   0.44129   0.00067  Uiso   1.00
O79    O    -0.01798   0.89093  -0.02213   0.00062  Uiso   1.00
O80    O     0.47454   0.74967   0.40029   0.00067  Uiso   1.00
O81    O     0.43156   1.08577   0.38160   0.00062  Uiso   1.00
Ru82   Ru    0.78158   0.52642   0.56042   0.00036  Uiso   1.00
Ru83   Ru    0.76473   0.32818   0.55969   0.00037  Uiso   1.00
Ru84   Ru    0.64161   0.41990   0.30014   0.00035  Uiso   1.00
C85    C     0.53872   0.53182   0.41280   0.00048  Uiso   1.00
C86    C     1.01903   0.51606   0.69965   0.00060  Uiso   1.00
C87    C     0.74934   0.64415   0.48011   0.00044  Uiso   1.00
C88    C     0.88830   0.55200   0.76175   0.00056  Uiso   1.00
C89    C     0.51987   0.33794   0.40799   0.00044  Uiso   1.00
C90    C     1.00709   0.32380   0.70750   0.00056  Uiso   1.00
C91    C     0.86066   0.31257   0.75589   0.00061  Uiso   1.00
C92    C     0.71848   0.20682   0.47954   0.00049  Uiso   1.00
C93    C     0.40109   0.42906   0.15659   0.00037  Uiso   1.00
C94    C     0.88412   0.40323   0.45093   0.00039  Uiso   1.00
C95    C     0.56755   0.31452   0.17342   0.00056  Uiso   1.00
C96    C     0.59581   0.52315   0.18611   0.00037  Uiso   1.00
O97    O     0.40075   0.54387   0.33438   0.00066  Uiso   1.00
O98    O     1.16582   0.51837   0.79295   0.00075  Uiso   1.00
O99    O     0.73009   0.71542   0.43130   0.00065  Uiso   1.00
O100   O     0.95656   0.56846   0.88458   0.00105  Uiso   1.00
O101   O     0.37629   0.33408   0.32075   0.00082  Uiso   1.00
O102   O     1.15073   0.31007   0.79762   0.00077  Uiso   1.00
O103   O     0.92105   0.30249   0.87532   0.00106  Uiso   1.00
O104   O     0.69263   0.13333   0.43162   0.00079  Uiso   1.00
O105   O     0.25254   0.42905   0.05871   0.00067  Uiso   1.00
O106   O     1.01798   0.39093   0.52213   0.00062  Uiso   1.00
O107   O     0.52546   0.24967   0.09971   0.00067  Uiso   1.00
O108   O     0.56844   0.58577   0.11840   0.00062  Uiso   1.00
loop_
_geom_bond_atom_site_label_1
_geom_bond_atom_site_label_2
_geom_bond_distance
_geom_bond_site_symmetry_2
_ccdc_geom_bond_type
Ru1    Ru2    2.892   .    S
Ru1    Ru3    2.882   .    S
Ru1    C4     1.953   .    S
Ru1    C5     1.945   .    S
Ru1    C6     1.909   .    S
Ru1    C7     1.928   .    S
Ru1    O18    3.068   .    S
Ru2    Ru3    2.887   .    S
Ru2    C8     1.957   .    S
```

Ru2	C9	1.946	.	S
Ru2	C10	1.924	.	S
Ru2	C11	1.907	.	S
Ru2	O23	3.065	.	S
Ru3	C12	1.953	.	S
Ru3	C13	1.945	.	S
Ru3	C14	1.909	.	S
Ru3	C15	1.923	.	S
Ru3	O27	3.078	.	S
C4	O16	1.156	.	S
C5	O17	1.158	.	S
C6	O18	1.158	.	D
C7	O19	1.155	.	D
C8	O20	1.155	.	S
C9	O21	1.156	.	S
C10	O22	1.155	.	D
C11	O23	1.158	.	D
C12	O24	1.155	.	S
C13	O25	1.157	.	S
C14	O26	1.156	.	D
C15	O27	1.155	.	D
Ru28	Ru29	2.892	.	S
Ru28	Ru30	2.882	.	S
Ru28	C31	1.953	.	S
Ru28	C32	1.945	.	S
Ru28	C33	1.909	.	S
Ru28	C34	1.928	.	S
Ru28	O45	3.068	.	S
Ru29	Ru30	2.887	.	S
Ru29	C35	1.957	.	S
Ru29	C36	1.946	.	S
Ru29	C37	1.924	.	S
Ru29	C38	1.907	.	S
Ru29	O50	3.065	.	S
Ru30	C39	1.953	.	S
Ru30	C40	1.945	.	S
Ru30	C41	1.909	.	S
Ru30	C42	1.923	.	S
Ru30	O54	3.078	.	S
C31	O43	1.156	.	S
C32	O44	1.158	.	S
C33	O45	1.158	.	D
C34	O46	1.155	.	D
C35	O47	1.155	.	S
C36	O48	1.156	.	S
C37	O49	1.155	.	D
C38	O50	1.158	.	D
C39	O51	1.155	.	S
C40	O52	1.157	.	S
C41	O53	1.156	.	D

C42	O54	1.155	.	D
Ru55	Ru56	2.892	.	S
Ru55	Ru57	2.882	.	S
Ru55	C58	1.953	.	S
Ru55	C59	1.945	.	S
Ru55	C60	1.909	.	S
Ru55	C61	1.928	.	S
Ru55	O72	3.068	.	S
Ru56	Ru57	2.887	.	S
Ru56	C62	1.957	.	S
Ru56	C63	1.946	.	S
Ru56	C64	1.924	.	S
Ru56	C65	1.907	.	S
Ru56	O77	3.065	.	S
Ru57	C66	1.953	.	S
Ru57	C67	1.945	.	S
Ru57	C68	1.909	.	S
Ru57	C69	1.923	.	S
Ru57	O81	3.078	.	S
C58	O70	1.156	.	S
C59	O71	1.158	.	S
C60	O72	1.158	.	D
C61	O73	1.155	.	D
C62	O74	1.155	.	S
C63	O75	1.156	.	S
C64	O76	1.155	.	D
C65	O77	1.158	.	D
C66	O78	1.155	.	S
C67	O79	1.157	.	S
C68	O80	1.156	.	D
C69	O81	1.155	.	D
Ru82	Ru83	2.892	.	S
Ru82	Ru84	2.882	.	S
Ru82	C85	1.953	.	S
Ru82	C86	1.945	.	S
Ru82	C87	1.909	.	S
Ru82	C88	1.928	.	S
Ru82	O99	3.068	.	S
Ru83	Ru84	2.887	.	S
Ru83	C89	1.957	.	S
Ru83	C90	1.946	.	S
Ru83	C91	1.924	.	S
Ru83	C92	1.907	.	S
Ru83	O104	3.065	.	S
Ru84	C93	1.953	.	S
Ru84	C94	1.945	.	S
Ru84	C95	1.909	.	S
Ru84	C96	1.923	.	S
Ru84	O108	3.078	.	S
C85	O97	1.156	.	S

C86	O98	1.158	.	S
C87	O99	1.158	.	D
C88	O100	1.155	.	D
C89	O101	1.155	.	S
C90	O102	1.156	.	S
C91	O103	1.155	.	D
C92	O104	1.158	.	D
C93	O105	1.155	.	S
C94	O106	1.157	.	S
C95	O107	1.156	.	D
C96	O108	1.155	.	D

Table S5. Cartesian coordinates of the Gaussian geometry optimised structure of $Ru_3(CO)_{12}$ with D_{3h} symmetry imposed.

Center Number	Atom Type	Coordinates (Å) X	Y	Z
1	Ru	0.000000	1.686168	0.000000
2	Ru	-1.460265	-0.843084	0.000000
3	Ru	1.460265	-0.843084	0.000000
4	C	0.000000	1.656448	1.958372
5	C	0.000000	1.656448	-1.958372
6	C	1.521483	2.878496	0.000000
7	C	-1.521483	2.878496	0.000000
8	C	-1.434526	-0.828224	1.958372
9	C	-1.434526	-0.828224	-1.958372
10	C	-3.253592	-0.121605	0.000000
11	C	-1.732109	-2.756891	0.000000
12	C	1.434526	-0.828224	1.958372
13	C	1.434526	-0.828224	-1.958372
14	C	1.732109	-2.756891	0.000000
15	C	3.253592	-0.121605	0.000000
16	O	0.000000	1.809468	3.122676
17	O	0.000000	1.809468	-3.122676
18	O	2.426632	3.627172	0.000000
19	O	-2.426632	3.627172	0.000000
20	O	-1.567046	-0.904734	3.122676
21	O	-1.567046	-0.904734	-3.122676
22	O	-4.354539	0.287938	0.000000
23	O	-1.927908	-3.915111	0.000000
24	O	1.567046	-0.904734	3.122676
25	O	1.567046	-0.904734	-3.122676
26	O	1.927908	-3.915111	0.000000
27	O	4.354539	0.287938	0.000000
